# Disruption of Intestinal Homeostasis Through Altered Responses of the Microbial Community, Energy Metabolites, and Immune System in Zebrafish After Chronic Exposure to DEHP

**DOI:** 10.3389/fmicb.2021.729530

**Published:** 2021-10-04

**Authors:** Pan-Pan Jia, Muhammad Junaid, Guang-Yuan Xin, Yan Wang, Yan-Bo Ma, De-Sheng Pei

**Affiliations:** ^1^School of Public Health and Management, Chongqing Medical University, Chongqing, China; ^2^Chongqing Institute of Green and Intelligent Technology, Chinese Academy of Sciences, Chongqing, China; ^3^Joint Laboratory of Guangdong Province and Hong Kong Region on Marine Bioresource Conservation and Exploitation, College of Marine Sciences, South China Agricultural University, Guangzhou, China

**Keywords:** chronic exposure, zebrafish development, gut microbiota, metabolic disturbance, DEHP

## Abstract

Di-(2-ethylhexyl) phthalate (DEHP) is ubiquitously reported in global water bodies and exhibits various environmental and human health risks. However, the effects of DEHP chronic exposure on the intestinal microbiota and associated host health concerns in aquatic species are still largely unexplored. In this study, chronic exposure to DEHP at environmental levels significantly increased the body weight, length, and body mass index (BMI), especially in male fish. The microbial community was disrupted with the relative abundance of phylum *Firmicutes* and genera diversity for *Prevotella-7*, *Deefgea*, *PeM15*, *Halomonas*, *Akkermansia*, *Chitinibacter*, and *Roseomonas*, which are significantly activated in zebrafish after exposure to DEHP. The height of the gut villus, the thickness of muscularis layer, and the number of goblet cells *per* villus were significantly decreased, as well as showed differences between female and male zebrafish. Further, the levels of energy-related metabolites in gut tissues were increased, compared to the control group. The expression levels of immune-related genes (interleukin 8, *il-8*, also referred to as *cxcl8a*), microbial defense-related genes (lysozyme, *lyz*, interleukin 10, and *il-10*), and obesity-related genes (aquaporin 8a, *aqp8*, mucin 2.1, *muc2.1*, fibroblast growth factor 2, *fgf2*, and proopiomelanocortin a, *pomca*) were significantly up-regulated in zebrafish, except the down-regulated expressions of toll-like receptor-5 (*tlr-5*) and interleukin 1β (*il-1*β) in the females and *pomca* in the males, respectively. Importantly, Spearman’s correlation analyses revealed that the levels of metabolites and gene expressions in the gut were closely related to the dominant microbial genera, such as *Aeromonas*, *Deefgea*, *Akkermansia*, *PeM15*, *Mycobacterium*, and *Rhodobacter*. Taken together, chronic exposure to DEHP at environmental levels disturbed bacterial composition accompanied by the altered expressions of intestinal metabolites and the critical immune and intestinal function-related genes, which provided novel insights into DEHP effects on perturbation of gut microbiota and metabolic homeostasis in zebrafish.

## Introduction

Phthalate esters (PAEs) are widely used as plasticizers in a great deal of domestic and industrial products, which leads to their inevitable release into the environment and exposure to humans and animals (Erythropel et al., 2014). Comprising approximately 50% of the total plasticizer products, di(2-ethylhexyl) phthalate (DEHP) is the most extensively used PAE, and its toxicity has attracted huge attention worldwide (Lyche et al., 2009; Rowdhwal and Chen, 2018). However, researches mostly focused on the potential risks of DEHP on neurodevelopment in early life, endocrine disruption, and reproductive toxicity (Ma et al., 2018a; Radke et al., 2018). The guidelines for DEHP in drinking water from World Health Organization [WHO] (2011) and U.S. Food and Drug Administration [U.S. FDA] (2012) have established maximum allowable concentrations in drinking water of 8 and 6 μg/L, respectively, above which the well-being of aquatic organisms and human health may be endangered. Recently, the environmental contamination of DEHP have been reported with various levels from 0.33 to 98.87 μg/L in the North Rhine River (Germany), as high as 2306 μg/L in the Eastern Cape River (South Africa), 1390 μg/L in Kunming Lake (China), and 6.1–94.1 μg/L in bottled waters from several countries (China, Saudi Arabia, Czech Republic, Croatia, and Thailand). Moreover, DEHP has been reported with elevated levels in various riverine systems worldwide (Fromme et al., 2002; Yuwatini et al., 2006; Magdouli et al., 2013; Junaid et al., 2018; Luo et al., 2018). DEHP, being a small molecule additive, is more prone to contaminate aquatic environments and induce ecological risks. Therefore, more investigations should be carried out to reveal its comprehensive toxicity, especially at environmental levels on aquatic organisms.

Environmental contaminants can disrupt gut microbiota, especially in aquatic species (Jin et al., 2017). The healthy gut microbiota affects several functions in the host, including nutrient metabolism, xenobiotic and drug metabolism, maintenance of structural integrity of gut mucosal barrier, immunomodulation, and protection against pathogens (Jandhyala et al., 2015; Marchesi et al., 2016). Moreover, the host-microbiota interactions are involved in the regulation of multiple metabolic functions, energy balance, and immune-inflammatory signaling that physiologically connect gut organs (Nicholson et al., 2012; Nieuwdorp et al., 2014). In fish, the intestinal bacterial communities are sensitively shaped by environmental and ecological factors (Sullam et al., 2012). Therefore, the composition of the microbial community is considered an important index to evaluate the toxicity of pollutants, which can be detected by modern biotechnology technique 16S rRNA high throughput sequencing.

Zebrafish model possesses numerous advantages such as short life span, transparent embryos, high similarity to the human genome, and diverse adaptability, thus credibly applied to evaluate the toxicity of various environmental pollutants, including DEHP (Dai et al., 2014; Jia et al., 2016). Moreover, zebrafish have been employed to study the functions and impacts of various pollutants on gut microbiota after acute or chronic exposure (Cheesman and Guillemin, 2007; Cantas et al., 2012; Ma et al., 2018b). Although the desorption of DEHP was reported in the fish gut (Coffin et al., 2018), the potential disruption of gut microbiota during chronic exposure in aquatic organisms is still elusive.

The toxicity assessment of PAEs showed their ability to affect immune and allergic responses, body weight, and intestinal development in experimental animals and *in vitro* (Kimber and Dearman, 2010; Ahmed et al., 2018). Previous reports highlighted that the utility and intestinal biology to functionally validate candidate genes identified through genome-wide association studies are significant to the zebrafish model (Zhao and Pack, 2017). A significant degree of homology has been reported between zebrafish intestines and that of higher vertebrates in terms of cellular composition and function. Moreover, the intestine acts as a digestive and immune organ with highly conserved molecular pathways, injury regulating functions, and immune responses. Therefore, deciphering the changes in bacterial community structure, the content of gut metabolites, and the genetic expressions of various signaling pathways could provide deep insights into the comprehensive toxicity of DEHP on fish developmental and associated health effects. Previously, chronic exposure to DEHP induced significant genotoxicity *via* pathways related to obesity and reproduction with potential adverse health implications (Wassenaar and Legler, 2017; Ma et al., 2021). Researchers have investigated a battery of immunity-related genes, such as toll-like receptor 5 (*tlr-5*), interleukin 1β (*il-1β*), nuclear factor κB (*nf-κb*), and interleukin 8 (*il-8* and *cxcl8a*) to interpret the immune system and intestinal functions in zebrafish (Udayangani et al., 2017; Arias-Jayo et al., 2018). Previous reports proved that the host bacterial handling and antimicrobial defense-related genes including nucleotide oligomerization domain 2 (*nod2*), lysozyme (*lyz)*, tumor necrosis factor-α (*tnf-α*), and interleukin 10 (*il-10*) were activated in responding to intestinal microbiota dysbiosis (Dotti et al., 2017; Arias-Jayo et al., 2018; Imhann et al., 2018). The crucial genes of aquaporin 8a (*aqp8*), mucin 2.1 (*muc2.1*), fibroblast growth factor 2 (*fgf2*), and proopiomelanocortin a (*pomca*) can indicate the intestinal secretory and absorptive-, and obesity-related functional abnormality in the host (Campion et al., 2009; Dotti et al., 2017; Montalbano et al., 2018). However, after chronic DEHP exposure, the inner mechanism has not been clearly studied yet to understand the toxic effects, especially the genetic and bacterial markers related to development, metabolism, and intestinal functions in aquatic species.

Thus, wild type zebrafish were employed as *in vivo* model in this study to explore the possible toxicity of chronic (3.5 months, from embryo to adult) DEHP environmental relevant exposure (0, 10, 33, and 100 μg/L) on development, gut microbiota, energy metabolism, and immune system responses. The disruption effects on the diversity and richness of gut microbiota in female and male fish were explored by 16S rRNA sequencing analysis. Moreover, DEHP-mediated damages on zebrafish gut tissues were detected using histomorphology analyses (villi and goblet cells indexes). The metabolites in zebrafish intestines related to the energy cycle were also measured to investigate the underlying relationships of the gut microbiota and metabolic homeostasis. Importantly, the mRNA levels of genes related to host immune response and antibacterial defense, and intestinal functions were also quantified. Last but not the least, the potential relationship between microbial community structure and functionally related genes in the gut was unveiled for both female and male zebrafish.

## Materials and Methods

### Ethics Statement

Zebrafish experiments were performed according to the “Guide for the Care and Use of Laboratory Animals” (Eighth Edition, 2011. ILARCLS, National Research Council, Washington, DC, United States). The animal protocol was approved by the Animal Care and Use Committee of Chongqing in China and by the Institutional Animal Care and Use Committee of Chongqing Institute of Green and Intelligent Technology, Chinese Academy of Sciences (Approval ID: ZKCQY0063).

### Chemicals

The analytical standard of DEHP (99.6%, No: ALR-097N) was purchased from AccuStandard (New Haven, CT, United States) and dissolved in dimethyl sulfoxide (DMSO, CAS: 67-68-5, purity ≥99.5%, Sigma-Aldrich, United States). Then the DEHP stock solution was stored at −20°C. 3-Aminobenzoic acid ethyl ester or methane-sulfonate salt (MS-222, 98%) was obtained from Aladdin (Los Angeles, California, United States). The triglyceride assay kit (No. A110-1), the pyruvate assay kit (No. A081), the non-esterified fatty acids kit (No. A042-2), and the glucose assay kit (No. F006) were purchased from Jiancheng Bioengineering Institute, Nanjing, China^1^. All other chemical reagents used in this study were of analytical grade.

### Zebrafish Maintenance, Chronic Exposure to DEHP, and Measurement of Developmental Toxicity

The maintenance of zebrafish (Danio rerio, AB strain) was described in our previous study (Jia et al., 2016). Briefly, adult zebrafish were maintained in flow-through systems with the condition of temperature (28 ± 0.5°C) and 14:10 h (light: dark) photoperiod and fed twice every day with the newly hatched brine shrimp (*Artemia nauplii*). Healthy adult zebrafish were placed in the breeding box with a ratio of 2:1 (male: female) overnight, and the spawning was triggered under light and completed within 30 min.

According to experimental design (Supplementary Figure 1), zebrafish embryos with normal development were selected and randomly distributed into glass beakers containing different concentrations of DEHP exposure solution (0, 10, 33, and 100 μg/L) with three replicates for each group. A total of 150 embryos were cultured in 200 mL volume of each glass beaker till developed to larvae, then the grown juvenile fish were cultured in 2 L glass tanks till the adult zebrafish changed to 10 L volume tanks. The corresponding DEHP stocked solution added in treated groups were A = 8 × 10^7^ μg/L, B = 8 × 10^6^ μg/L, C = 8 × 10^5^ μg/L, and the same volume of DMSO (<0.003%) was used as the control group. During the experimental process, half of the exposure solution in each group was renewed daily and zebrafish were maintained and fed twice with enough egg yolk (2–4 mL) for the larvae (7–14 days post-fertilization, dpf) and newly hatched brine shrimp (12–30 mL) for the juvenile (14–28 dpf) to adult ones (28 dpf–3.5 months post-fertilization, mpf). After exposure for 3.5 months, the adult zebrafish were starved for 12 h and washed with ultrapure water before being anesthetized in 100 mg/L of MS-222, and then dissected to collect entire intestines and other tissues. The extracted samples were immediately frozen into liquid nitrogen and stored at −80°C for the subsequent analysis.

After exposure experiments, the developmental indices of adult zebrafish including body weight, body length from head to tail, and organ weight of intestines, gonad, and liver tissues were recorded. The body mass index [BMI, body weight(mg)/body length(cm)^2^], coefficients of Fulton’s condition factor [K, body weight(g)^∗^100/body length(cm)^3^], and the organ weight ratio [organ weight(g) × 100%/body weight(g)] of intestines, brain, liver, and gonad tissues was calculated to evaluate chronic effects of DEHP exposure on the development of both male and female zebrafish, following the previously described method (Clark et al., 2018).

### The 16S rRNA Sequencing and Taxonomic Analysis and Composition of Zebrafish Intestinal Microbiota

The intestinal microbiota of male and female zebrafish was detected *via* the high-throughput sequencing with three replicate entire gut tissues from each group, and analysis methods were performed according to our previous report (Jia et al., 2019). After the total DNA extraction and quality measurement, the intestinal microbial community composition of zebrafish was detected by MiSeq Illumina PE300 platform (Illumina, San Diego, CA, United States) using adapter primers 338F: (ACTCCTACGGGAGGCAGCA) and 806R: (GGACTACHVGGGTWTCTAAT) for the V3-V4 regions of the 16S rRNA gene (methods shown in Supplementary Text 1 and detailed software in Supplementary Table 1). The original sequencing data of adult zebrafish in this study were stored at the NCBI database with Accession number: PRJNA615951.

The raw 16S rRNA gene sequencing reads were demultiplexed, quality-filtered by Fastp 0.19.6, and merged by Flash version 1.2.11 with the following criteria: the maximum number of errors in the barcode was 0 and the number of mismatches in the primer was 2. Reads with more than 10% of bases with a quality score of Q < 20, and ambiguous or unassigned characters and adapter contamination were removed. USEARCH (version 7.0^2^) was used to classify the operational taxonomic units (OTUs) based on sequence similarity by cluster cut-off value of 97%. The taxonomy of each OTU representative sequence was analyzed by RDP Classifier version 2.2 against the 16S rRNA database (Release 138^3^) using a confidence threshold of 0.7. The bacterial alpha diversity indexes were calculated by Mothur (version 1.30.2), and the alpha rarefaction curves showed that the reads number of each sample was sufficient for subsequent analysis.

The zebrafish intestinal community was analyzed based on the OTUs abundance in each sample with the Good’s coverage and different alpha diversity indexes. Among them, Shannon and Simpson reflected the community diversity, while the abundance-based coverage estimator (Ace) and Chao1 indicated the community richness. Moreover, the composition of the phylum and genus with the community heatmap was analyzed in male and female fish. Additionally, the Partial Least Squares Discriminant Analysis (PLS-DA) analysis and Linear Discriminant Analysis (LDA) effect size (LEfSe) analysis (http://huttenhower.sph.harvard.edu/galaxy/root?tool_id=lefse_upload) were applied to deeply explore the major changes of intestinal bacterial between treatment and sex groups. Moreover, the MaAsLin (Multivariate Association with Linear Models) was carried out here to explore the relationship between environmental factors and microbial richness and functions. Finally, the bacterial functions in the zebrafish gut were predicted by PICRUSt software with EggNOG (evolutionary genealogy of genes: Non-supervised Orthologous Groups, http://eggnog.embl.de/) database and KEGG (Kyoto Encyclopedia of Genes and Genomes^4^) database.

### The Histomorphology Analysis of Zebrafish Intestines

The histomorphology of three replicate samples of exposed zebrafish intestine was performed from tissues immediately fixed in 4% (w/v) formaldehyde solution for 24 h, then dehydrated in ethanol, and embedded in paraffin. The zebrafish gut slices were stained with hematoxylin and eosin (H&E), and the analysis of villi height and width, and muscularis thickness were calculated according to the previous studies (Escaffre et al., 2007; Jia et al., 2019). Moreover, the gut slices were stained with Alcian Blue-Periodic Acid Schiff (AB-PAS) to check the number of goblet cells and morphometric parameters at the 200 × magnification by photographed with a digital microscope (Nikon, SMZ18) with a scale marked with NIS-Elements imaging software (version 4.30).

### The Detection of Energy-Related Metabolites in Zebrafish Intestines

The triglyceride (TG), pyruvate (PY), glucose (Glu), and non-esterified fatty acids (FA) contents in zebrafish entire gut tissues were measured using assay kits according to the manufacturer’s instructions. The steps to detect the TG, PY, FA, and Glu levels can be seen in Supplementary Text 2, and the contents of TG, PY, FA, and Glu were calculated by the following formulas:


TGcontent=[(OD-sampleOD)blank/(OD-standardOD)blank]*

$${\text{Con}}_{\text{standard}}/{\text{Con}}_{\text{sample}}$$

PYcontent=[(OD-sampleOD)blank/(OD-standardOD)blank]*

$${\text{Con}}_{\text{standard}}/{\text{Con}}_{\text{sample}}$$

FAcontent=[(ΔA-sampleΔA)blank/(ΔA-standardΔA)blank]*

$${\text{Con}}_{\text{standard}}/{\text{Con}}_{\text{sample}}$$

Glucontent=[(OD-sampleOD)blank/(OD-standardOD)blank]*

$${\text{Con}}_{\text{standard}}/{\text{Con}}_{\text{sample}}$$

### The Expression Profiling of Immune-Related and Functional Genes in Zebrafish Intestines

For the analyses of gene expression, the total RNA was extracted from zebrafish gut tissues, and three intestines of the same sex from one tank were pooled together as one biological sample of three replicate samples (*n* = 3) in each group, using RNAiso Plus reagent (Takara, Japan) according to the manufacturer’s instructions. High-quality RNA samples were prepared for cDNA using the Prime Script Reverse Transcription Reagent kit (Takara, China), and quantitative real-time PCR (qRT-PCR) was performed using ABI 7500 system (Applied Biosystems, Waltham, MA, United States) with SYBR^®^ Premix Ex Taq^TM^ II (Takara, Japan). The primers for selected zebrafish genes were designed from NCBI Primer-BLAST^5^, and the primer sequences were shown in Table 1. Running program initiated from 95°C, incubated for 5 min, and followed by 40 cycles of 95°C for 15 s, 58° for 30 s, and 72°C for 45 s. Three independent biological replicates were performed in each group, and the 2^–ΔΔ*CT*^ method was used to quantify relative mRNA expression levels.

**TABLE 1:** The primers of related genes in zebrafish in this study.

Name	F(5′-3′)	R(5′-3′)	Gene No.	Product Length (bp)
*gapdh*	GTGGAGTCTACTGGTGTCTTC	GTGCAGGAGGCATTGCTTACA	NM_001115114.1	173
**Functions: key genes in the intestinal immune system**				
*tlr-5*	GAAACATTCACCCTGGCACA	CTACAACCAGCACCACCAGAATG	NM_001130595.2	70
*il-1β*	CCACGTATGCGTCGCCCAGT	GGGCAACAGGCCAGGTACAGG	NM_212844.2	91
*nf-κb*	AACGAGGCTGAGAGGAGTCT	ATCAGCCGAACCACACTGAG	NM_001353873.1	80
*il-8 (cxcl8a)*	GCTTCTGATCTGCACGACTG	CCACGTCTCGGTAGGATTGA	NM_001327985.1	220
**Functions: host bacterial handling and antimicrobial defense**				
*nod2*	ACACCCTCACAACAGGTTCG	ATTGGTGAGGTTGGCTCAGG	NM_001328044	110
*lyz*	CGTGGATGTCCTCGTGTGAA	TAGGCCGTGCACACATAGTT	NM_139180.1	121
*tnf-α*	TCACGCTCCATAAGACCCAG	AATGGATGGCAGCCTTGGAA	NM_212859.2	108
*il-10*	CGGGATATGGTGAAATGCAAGA	AGAGCAAATCAAGCTCCCCC	NM_001020785	130
**Functions: intestinal secretory and absorptive, obesity related genes**				
*aqp8*	CAGGAGCAGCGTTTAATGCC	TGATATTCCTCCCCCAGCCA	NM_001004661	194
*muc2.1*	GCAAGACTTGTGGCCTTTGT	AGCTGTCTGGATTGACCGTG	XM_021470771	264
*fgf2*	AGCGCCTGTACTGCAAAAAC	CTGTGGTCCTTTTCGTCCCG	NM_212823.2	207
*pomca*	GTGGGTTTCCTACCTGCACA	CCCCTCACCATCTCTTTTGC	NM_181438.3	205

### Statistical Analysis

The statistical analysis was performed using the Kolmogorov-Smirnov test and Leven’s test to define the normality of data and the homogeneity of variance. The differences between the variables were calculated by *t*-test and one-way analysis of variance (ANOVA), followed by Dunnett’s test using SPSS 20.0 software (SPSS, Chicago, IL, United States). Spearman’s correlation and Pearson’s correlation analyses were performed between the levels of metabolites, intestinal functions-related genes, and major genera in zebrafish. The correlation was analyzed by SPSS with a two-tailed test and the 95% confidence interval, and indicated by a color gradient based on correlation coefficient values. The value of *p* < 0.05 was denoted as statistically significant differences, and all values were presented as the means ± standard error (SEM) of the replicates in each group.

## Results

### Developmental Effects of DEHP Chronic Exposure in Zebrafish

**FIGURE 1:**
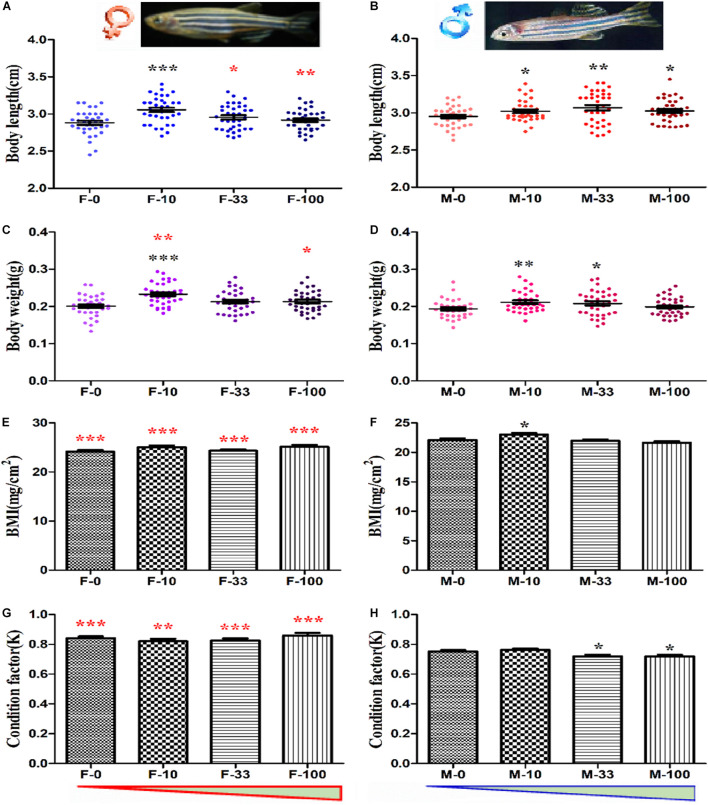
The developmental indexes of zebrafish after long-term DEHP exposure. The body length (cm) of female **(A)** and male **(B)** fish, and the body weight (g) of female **(C)** and male **(D)** fish were presented. The BMI and condition factor (K) of female **(E,G)** and male **(F,H)** fish were calculated. The data were presented as the mean ± standard error (SEM) of (12 pairs recorded/replicate, 3 biological replicates/group, *n* = 36 female and 36 male) zebrafish in each group. The black symbols *, **, and *** showed *p* < 0.05, 0.01, and 0.001 with significant differences between exposure and control groups (F-0 vs. F-10, F-0 vs. F-33, F-0 vs. F-100, M-0 vs. M-10, M-0 vs. M-33, and M-0 vs. M-100), and the red symbol *, **, and *** stands for the significances between female and male in the same groups (F-0 vs. M-0, F-10 vs. M-10, F-33 vs. M-33, and F-100 vs. M-100). The red and blue triangles are represented for the concentration trend of DEHP exposure in female and male groups.

The chronic exposure to DEHP at environmentally relevant concentrations induced no obvious toxicity in the early life stages on the survival and growth of zebrafish (Supplementary Figure 2). However, the DEHP exposure from embryos to adult stages significantly altered the basic developmental indexes (Figure 1). For instance, the average body length was significantly increased (*p* = 0.035–0.0001) to 3.056 ± 0.029 cm for female in the lowest concentration group (10 μg/L) and 3.021 ± 0.023 cm, 3.068 ± 0.036 cm, 3.026 ± 0.025 cm for male in the 10, 33, and 100 μg/L groups, compared to the corresponding control groups for female (2.881 ± 0.027 cm) and male (2.951 ± 0.022 cm) (Figures 1A,B). Similarly, the body weight was significantly increased (*p* = 0.035–0.0001) to 0.233 ± 0.005 g in 10 μg/L exposed female zebrafish, and 0.210 ± 0.005 g, 0.207 ± 0.006 g in the 10 and 33 μg/L DEHP exposed male zebrafish, compared to control female (0.201 ± 0.005 g) and male (0.193 ± 0.004 g), respectively (Figures 1C,D). Moreover, the body mass index (BMI, mg/cm^2^) of male fish was significantly increased (*p* = 0.019) (23.028 ± 0.27) at 10 μg/L groups, while in other female and male groups, a moderate increase without significant changes was observed, compared to control groups (Figures 1E,F). However, the condition factor (K, g^∗^100/cm^3^) in male zebrafish was significantly decreased (*p* = 0.035 and 0.026) after 33 and 100 μg/L DEHP exposure, compared to controls (Figures 1G,H). Among the indexes, the body length of F-33 and F-100, and body weight of F-10 and F-100 were significantly different (*p* = 0.021–0.0001) compared to that of M-10, M-33, and M-100 groups, and the BMI and K factor of females were dramatically higher than that of male fish (*p* = 0.005–0.0001) (Figures 1A–H). For the organic weight ratio in zebrafish, the intestinal somatic index (ISI) of female and male was increased to 5.300 ± 0.227 and 3.584 ± 0.161 in the DEHP exposed groups but showed no significant changes, compared to female (4.706 ± 0.249) and male (3.334 ± 0.215) in the control group (Supplementary Table 2). Similarly, the other index, including gonadal somatic index (GSI) and hepatosomatic index (HSI), gently increased but showed no significant changes between DEHP treatment and control groups (Supplementary Table 2). However, the female fish exhibited significantly higher ISI and HSI than the male with *p* values ranged from 0.009 to 0.0001, except the GSI with no comparison.

### Composition of Zebrafish Intestinal Microbiota

The intestinal microbiota of zebrafish in control and the highest concentration of DEHP exposure (100 μg/L) groups were analyzed *via* 16S rRNA sequencing. The taxonomic result of total samples was 18 Phyla, 34 Classes, 87 Orders, 144 Families, 247 Genera, 324 Species, and 406 OTUs. Similar to our previous studies (Ma et al., 2018b; Jia et al., 2019), the composition and metabolism pathways of intestinal microbiota in female and male zebrafish were different. Thus, the following analysis of each group was departed by sex, marked by female and male in control (F-0 and M-0), female and male in 100 μg/L DEHP group (F-100 and M-100).

The rarefaction curves of OTUs showed that all samples reached the plateau phase by 26,000 sequence reads (Supplementary Figure 3), and the rarefaction curves of the Shannon index approached the plateau from about 4200 sequences per sample (Supplementary Figure 4). In general, we obtained 913,364 high-quality sequences and 400,891,017 bp bases with an average length of 438.917 bp, and the OTUs in each group were optimized sequence reads for the subsequent analysis. Moreover, the Good’s coverage index of samples was higher than 99.9% in all groups, indicating that the range of detected data was high and credible, and the unmeasured part was negligible. The diversity (Shannon, Simpson) and richness (Chao, Ace) indexes were calculated without significant differences by *t*-test between control and the DEHP exposed groups. The Shannon index in the M-100 treatment group was significantly changed, compared to the M-0 control group. Although the alteration in the diversity and richness of intestinal microbiota differed in female and male zebrafish, there was no statistical significance. In the F-100 and M-0 group, the Chao (263.55 ± 17.09 and 209.74 ± 18.86) and Ace (261.28 ± 20.81 and 218.87 ± 14.24) index values were higher than the F-0 and M-100, respectively, indicating the higher diversity in DEHP exposed female and lower diversity in DEHP exposed male. Consistent with other indexes, the lower Shannon values (2.35 ± 0.36 and 1.87 ± 0.29) and the higher Simpson index (0.21 ± 0.04 and 0.30 ± 0.07) highlighted the lower diversity in the F-100 group and M-0 group (Supplementary Table 3).

### Changes in Bacterial Community Structure in Zebrafish Intestines

**FIGURE 2:**
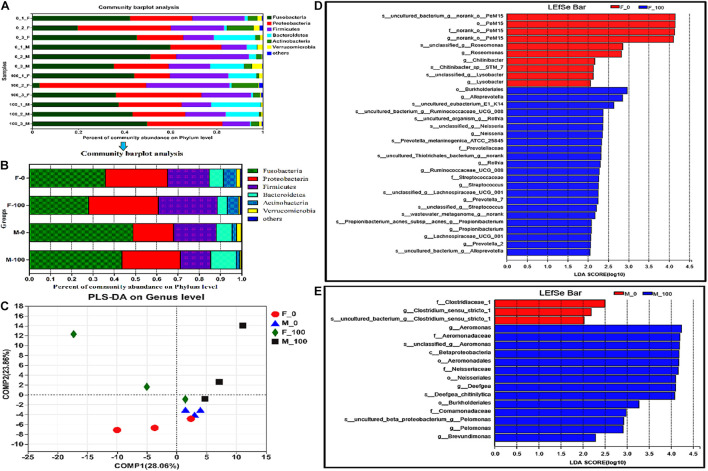
The analysis of zebrafish intestinal microbiota community at levels from phylum to species. The composition of intestinal microbiota at the phylum level (distribution in all samples **A** and averaged in groups **B**), and PLS-DA analysis **(C)** in zebrafish after DEHP exposure, and the average value of three replicates (*n* = 3) for female and male samples in each group was calculated, and the value of phylogenetic groups accounting for < 0.01 was summarized as “others.” The LEfSe analysis of intestinal microbiota showed the major affected bacteria in female **(D)** and male **(E)** zebrafish from phylum to species level, with the LDA value >2 and all-against-all (stricter) strategy.

The bacterial community structure and composition differ greatly at the phylum level in the intestines of both female and male zebrafish, between control and DEHP treatment groups (the distribution of phyla in all samples shown in Figure 2A, and the average of replicate samples from groups shown in Figure 2B). In the taxonomy analysis, the major dominant bacterial phyla in the F-0 group were *Fusobacteria* (35.85 ± 8.08%), *Proteobacteria* (29.16 ± 5.98%), *Firmicutes* (19.69 ± 3.11%), *Bacteroidetes* (6.77 ± 5.52%), and *Actinobacteria* (6.02 ± 2.91%). In the F-100 group, the abundances of *Fusobacteria* (27.94 ± 12.57%), *Bacteroidetes* (4.62 ± 3.73%), and *Actinobacteria* (5.23 ± 3.17%) were decreased, while for phyla *Proteobacteria* (32.89 ± 9.10%) and *Firmicutes* (27.73 ± 4.39%) were increased. Nevertheless, the percentages of dominant bacterial phyla in the M-0 group were in the following order: *Fusobacteria* (48.79 ± 7.18%), *Proteobacteria* (19.07 ± 3.95%), *Firmicutes* (20.15 ± 6.06%), *Bacteroidetes* (7.50 ± 3.66%), and *Actinobacteria* (2.04 ± 0.92%). In the M-100 group, the abundances of *Fusobacteria* (43.64 ± 3.55%), *Firmicutes* (14.04 ± 1.75%), and *Actinobacteria* (1.53 ± 0.94%) were decreased, but in other bacterial phyla *Proteobacteria* (27.64 ± 2.79%), and *Bacteroidetes* (12.13 ± 6.09%) were increased (Figure 2B). Notably, the *Firmicutes* in the F-100 were significantly different from those in the M-100, even the major phyla in control female and male fish were not statistically different. The *Verrucomicrobia* in the F-100 (0.82 ± 0.39%) and M-100 (0.80 ± 0.16%) groups were lower than those in the F-0 and M-0 groups (1.90 ± 0.46%, 2.30 ± 1.20%), respectively. Finally, the richness of minor bacterial phyla as “other” in the F-100 (0.77 ± 0.45%) and M-100 (0.21 ± 0.16%) zebrafish were increased, respectively, compared to the F-0 (0.60 ± 0.37%) and M-0 (0.14 ± 0.05%) control groups. In the PLS-DA analysis, the similarity at genus level of exposed female and male groups was distinguished from the corresponding control, but showed no statistical significance by PERMANOVA test with *p*-value 0.25 and R^2^ 0.31 (Figure 2C). Thus, the LEfSe analysis screened the major changed intestinal bacteria between treatment and sex groups from phylum to species levels with the LDA value >2 and all-against methods (Figures 2D,E). In exposed female groups, genera *Alloprevotella*, *Neisseria*, *Prevotella-7*, and *Streptococcus* were mainly affected owing to samples’ richness and diversity with LDA score 2.0–3.0 (Figure 2D). While in male zebrafish, fewer genera were considered to have huge effects on the microbial community with higher LDA scores of 2.5–4.5, such as *Aeromonas*, *Deefgea*, and *Pelomonas* (Figure 2E), indicating the differences between bacterial community responses in female and male fish to DEHP chronic exposure.

**FIGURE 3:**
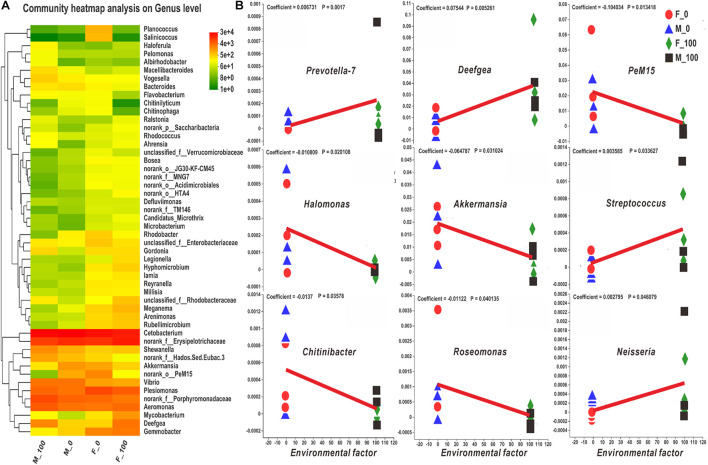
The heatmap and MaAsLin analysis of zebrafish intestinal bacteria according to the level of genus. **(A)** The bacterial hierarchical genera were clustered by the average manner, and the top 50 genera shown in the map were calculated by the log value of bacterial abundance in each group. **(B)** In the MaAsLin analysis of top 100 genera, the abundance of *Prevotella-7*, *Deefgea*, *PeM15*, *Halomonas*, *Akkermansia*, *Streptococcus*, *Chitinibacter*, *Roseomonas*, and *Neisseria* genera **(B)** were significantly related to the environmental factor of DEHP exposure with *p* < 0.05. In the heatmap, the X and Y axes are groups and genera with the cluster tree on the left, and the scale shown on the right side is the color range of different abundance values. In the MaAsLin legend, the X-axis means the relative abundance of each genus, and Y-axis indicates the environmental factor of DEHP exposure.

The intestinal bacteria in control and DEHP exposed groups were identified by the Venn diagram according to specific and shared family levels between female and male zebrafish (Supplementary Figure 5). Further, the intestinal microbiota was analyzed, and the community heatmap of the top 50 genera with the hierarchical clustering was calculated according to the average abundance value of the samples to investigate the potential effects of DEHP (Figure 3). The bacterial abundance (fold-changes) in female and male zebrafish intestines varied at the genus level after DEHP exposure, e.g., for *Deefgea* in the M-100, it was significantly differed compared to that of control, and for *g_norank_f_Erysipelotrichaceae*, *Aeromonas*, *Shewanella* were significantly differed in the F-100, compared to that in the M-100 fish (Supplementary Table 4 and Figure 3A). Among the most abundant genera, *Aeromonas*, *Deefgea*, *Shewanella*, *Mycobacterium*, *g_unclassified_f_Rhodobacteraceae*, and *Hyphomicrobium*, as well as lower richness genera *Macellibacteroides* and *Haloferula* in DEHP exposure groups were increased with 1.29-, 6.73-, 1.17-, 4.85-, 3.53- and 1.59-, 1.77- and 3.28-folds changes in female fish, and 1.62-, 4.53-, 2.02-, 4.37-, 5.10- and 2.02-, 2.76-, and 13.08-folds changes in male fish compared to the control groups. While for the *Cetobacterium*, *Akkermansia*, *g_norank_o_PeM15*, *Bacteroides*, *Rhodobacter*, *Planococcus*, and *Salinicoccus*, the abundance levels were decreased to 0.79-, 0.40-, 0.09-, 0.58-, 0.42-, 0.01- and 0.00-folds in female fish, and to 0.88-, 0.20-, 0.02-, 0.33-, 0.86-, 0.06- and 0.17-folds in male fish, compared to control group, respectively. Notably, the *Plesiomonas*, *g_norank_f_Porphyromonadaceae*, *g_norank_f_Hados.Sed.Eubac.3*, and *g_unclassified_ f_Enterobacteriaceae* genera were decreased in female fish with 0.71- to 0.37-folds but showed higher levels with 1.30- to 1.68-folds in male fish after DEHP exposure. In contrast, the abundance of *g_norank_f_Erysipelotrichaceae*, *Gemmobacter*, *Meganema*, and *Rubellimicrobium* genera were increased in female fish with 1.25- to 1.60- folds but showed lower levels with 0.87- to 0.23-folds in the exposed male fish. Here, DEHP exposure changed gut bacterial genus differed in female and male zebrafish, such as the *Gordonia* increased with 6.71- folds in male fish and *Legionella* increased with 3.86- folds in female fish without any change in abundance in male fish, respectively. The MaAsLin analysis revealed the relationship of the zebrafish microbial community to the environmental factor (i.e., DEHP exposure concentrations) (Figures 3B–G). Among the top 100 bacteria, the genera of *Prevotella-7*, *Deefgea*, *Streptococcus*, and *Neisseria* were closely related to DEHP exposure with positive coefficients and *p*-value < 0.05 (Figure 3B). However, the genera of *PeM15*, *Halomonas*, *Akkermansia*, *Chitinibacter*, and *Roseomonas* were correlated to DEHP exposure with negative coefficients and *p*-value < 0.05 (Figure 3B).

Further, the intestinal bacterial functions in DEHP exposed and control groups were analyzed from the databank of Bacteria and Archaea based on the abundance of COG metabolism pathways (Supplementary Figure 6). In those functional pathways, A: RNA processing and modification, I: Lipid transport and metabolism, N: Cell motility, and Q: Secondary metabolites biosynthesis, transport, and catabolism showed increased abundance in female and male fish, compared to control groups. Notably, the abundance of KEGG metabolic pathways at the first level showed that most pathways, including cellular process and metabolism pathways, also increased in both female and male fish after DEHP exposure (Supplementary Figure 7). Especially, the metabolic pathways at the second level involved in the xenobiotics biodegradation and metabolism, cell motility, metabolism of other amino acids, circulatory system, neurodegenerative diseases, metabolism of terpenoids and polyketides, and lipid metabolism signaling potentially increased with 1.27-, 1.12-, 1.13-, 1.23-, 1.19-, 1.19-, and 1.15- folds in female fish, and with 1.19-, 1.32-, 1.10-, 1.49-, 1.18-, 1.15-, and 1.12- folds in male fish, compared to controls (Supplementary Figure 8). Besides, the pathways of the digestive system were increased by 1.42- folds in male fish, and the cardiovascular diseases increased to 1.25- folds in female fish, indicating that several response pathways were affected by DEHP exposure.

### Histomorphology of Zebrafish Intestines

**FIGURE 4:**
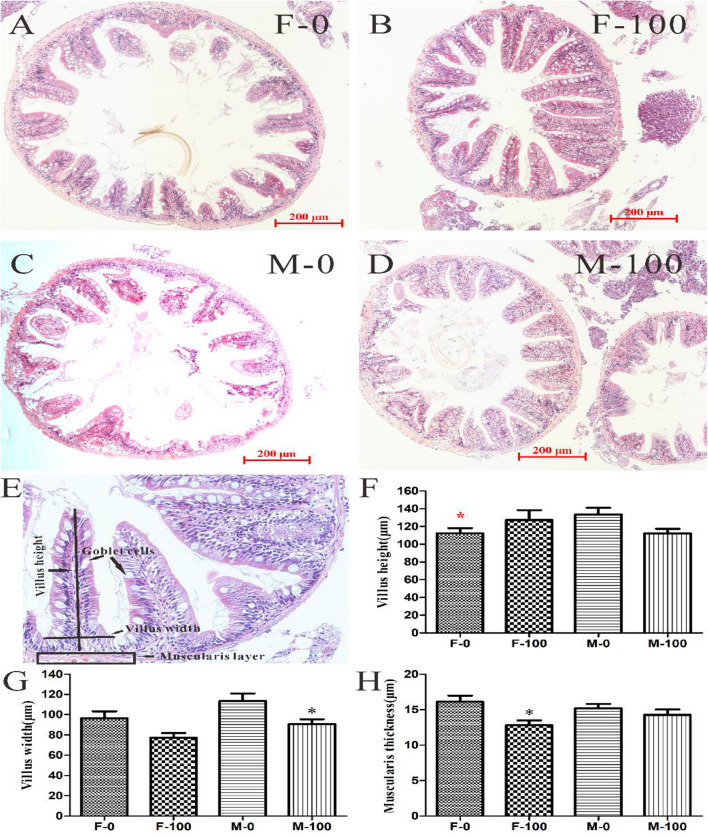
The histomorphological analysis of zebrafish intestine tissues with H&E stain. The histological analysis of gut tissue in female **(A)** and male **(C)** zebrafish in the control group, female **(B)** and male **(D)** zebrafish in 100 μg/L DEHP treatment group. According to the gut tissue indexes **(E)**, the height **(F)** and width **(G)** of villi, and muscular thickness **(H)** were measured from sections with 200 μm scale bar and 200 × images. And the symbol black * indicated *p* < 0.05 as the significant changes compared to control, the red * stands for the *p* < 0.05 as the significant changes of F-0 vs M-0.

For the histological analysis, the villi height, villi width, and muscularis thickness were detected in female and male zebrafish intestine sections with H&E staining (Figure 4). The intestine tissues were observed normally in the female and male controls (Figures 4A,C), and also no obvious damage was observed in DEHP exposed groups, compared to control (Figures 4B,D). The average villi height of intestine tissues was normal in the F-100 (127.32 μm) and M-100 (112.27 μm) exposed groups compared to their controls, respectively, but the F-0 (112.46 μm) was significantly lower (*p* = 0.026) than M-0 (133.54 μm) fish (Figure 4E). However, the average width of villi in the DEHP exposed male (90.60 μm) zebrafish intestines were significantly decreased (*p* = 0.011), compared to control group male (113.56 μm) zebrafish, while the female (77.14 μm) showed no significant changes compared to control group female (96.44 μm) (Figure 4E). Moreover, the average muscularis thickness of the F-100 and M-100 intestines was 12.81, and 14.28 μm, with the F-100 group, appeared significantly decreased (*p* = 0.015) compared to the control female zebrafish (16.12 μm) (Figure 4E).

**FIGURE 5:**
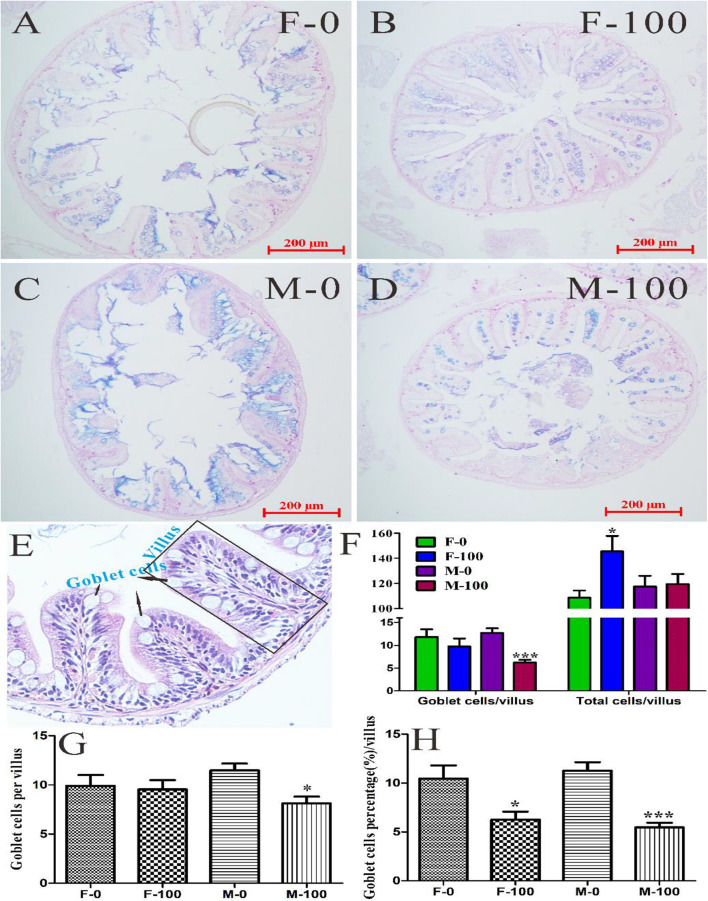
The goblet cells in zebrafish intestine tissues with AB-PAS and H&E stain. The histological analysis of gut tissue in female **(A)** and male **(C)** zebrafish in the control group, female **(B)** and male **(D)** zebrafish in the 100 μg/L DEHP treatment group. From the H&E sections of gut tissues **(E)**, the goblet cells and total cells of each villus **(F)** were counted. **(G)** The number of goblet cells was counted from AB-PAS sections with 200 μm scale bar and 200× images, and **(H)** the percentages of goblet cells *per* villus were calculated by comparing total cells in the corresponding villus. There was no difference between females and males in the same group, and the symbol * and *** indicated *p* < 0.05 and 0.001 as the significant changes compared to control.

To explore the effects of chronic exposure of DEHP on zebrafish intestinal functions, the AB-PAS staining of goblet cells was performed in this study (Figure 5). The goblet cells were counted in the intestine tissues of female and male zebrafish from both the control group (Figures 5A,C) and DEHP exposed group (Figures 5B,D). Besides goblet cells, the total number of cells in each villus was counted from zebrafish gut tissues H&E sections and found that the total cells, including primarily the epithelial cells and leukomonocyte, were significantly changed (*p* = 0.010) in the F-100 group with 145.39 cells per villus. At the same time, the goblet cells in the M-100 group were significantly reduced (*p* < 0.0001) to 6.22 cells per villus compared to the M-0 group with 12.70 cells (Figures 5E,F). The average number of goblet cells per villus with AB-PAS stain in the F-100 and M-100 group (9.5 and 8.1 cells) was decreased, compared to F-0 (9.9 cells) and M-0 (11.5 cells) control groups (significance level for M-100, *p* = 0.011), which was consistent with the goblet cells trend in H&E sections (Figure 5G). With comparison to total cells in the corresponding villus, the percentages of goblet cells per villus were significantly decreased (*p* = 0.011 and <0.0001) with 6.27 and 5.47%, respectively, in female and male zebrafish by DEHP exposure, compared to control groups (10.45 and 11.26%) (Figure 5H), indicating the potential negative effects on gut tissues and associated functions.

### Levels of Metabolites and Genes Expressions in Zebrafish Intestines

**FIGURE 6:**
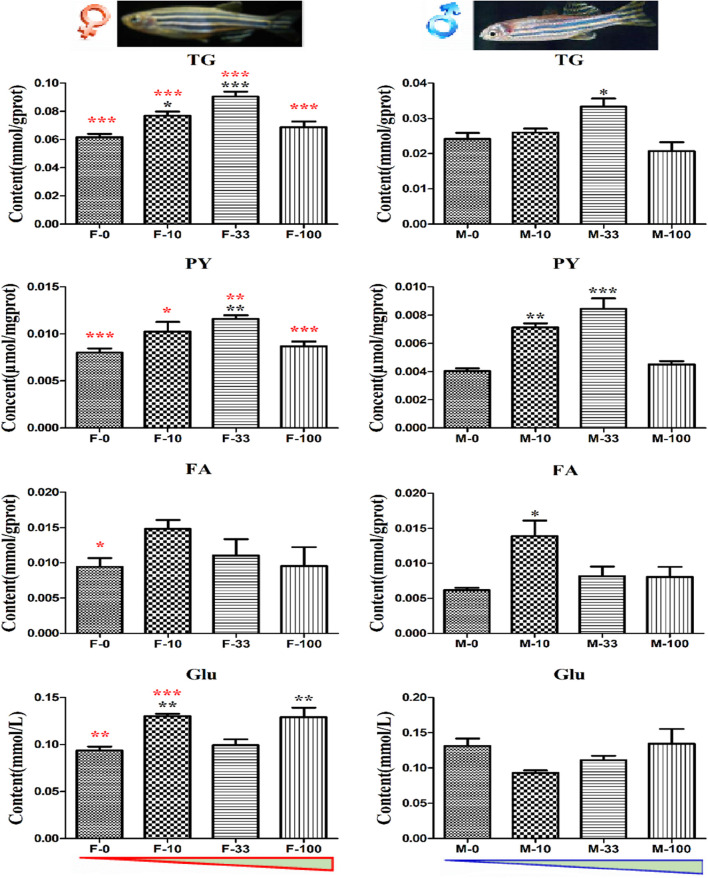
The energy metabolites in DEHP exposed zebrafish intestines. The contents of metabolites TG, PY, FA, and Glu related to the energy cycle in treated zebrafish were measured with three biological replicates *per* group, and the black symbols *, **, and *** implied *p* < 0.05, 0.01, and 0.001, respectively. Moreover, the contents in both sex zebrafish were different, and the red color symbols *, **, and *** stand for the significant difference between female and male fish in the same groups. The red and blue triangles mean the increasing DEHP exposure concentration in female and male groups.

The metabolites in zebrafish intestine tissues related to energy balance and body obesity were measured, and the TG content was significantly increased (*p* = 0.011–0.0001) in the F-10, F-33, and M-33 groups at the levels of 0.077, 0.091, and 0.033 mmol/gprot, compared to the F-0 and M-0 groups (0.062 and 0.024 mmol/gprot), respectively (Figure 6). Moreover, the TG content in female zebrafish intestines was significantly higher (*p* < 0.001) than males in the control and all exposed groups, respectively. Similarly, the PY was also increased (*p* = 0.0005–0.0001) in the F-33, M-10, and M-33 groups by 0.012, 0.007, and 0.008 μmol/mgprot, compared to the F-0 and M-0 groups (0.008 and 0.004 μmol/mgprot), respectively (Figure 6). It was also showed the sex difference of PY content in the female significantly higher (*p* = 0.0107–0.0001) than the male in control and all treated corresponding groups. The level of FA in females was significantly higher (*p* = 0.03) than males in control, but a significant difference (*p* = 0.015) was observed only in the M-10 group with FA levels of 0.014 mmol/gprot, compared to the M-0 group (0.006 mmol/gprot) (Figure 6). Interestingly, the Glu content was also significantly enhanced (*p* = 0.008 and <0.0001) to 0.130 and 0.129 mmol/L in the F-10 and F-100 groups, compared to 0.094 mmol/L in the control group. And the Glu content in F-0 and F-10 was significantly different (*p* = 0.003–0.0001) with M-0 and M-10 groups. However, the Glu content appeared with no changes in male zebrafish after DEHP exposure.

**FIGURE 7:**
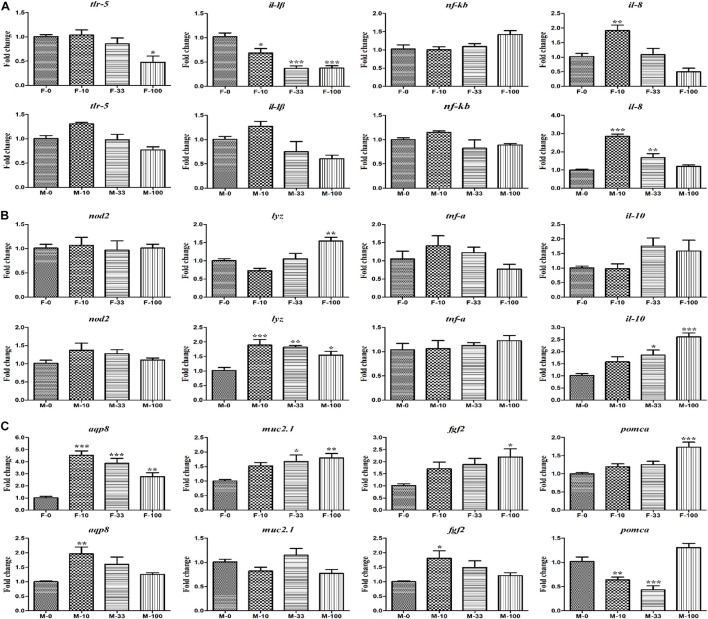
The expression of the intestinal functions-related genes in DEHP exposed zebrafish intestines. **(A)** The relative expression of *tlr-5*, *il-1β*, *nf-κb*, and *il-8* genes involved in the immune response. **(B)** The expression of *nod2*, *lyz*, *tnf-α*, and *il-10* genes related to bacterial handling and antimicrobial defense. **(C)** The secretory and absorptive, obesity-related genes of *aqp8*, *muc2.1*, *fgf2*, and *pomca* in female and male zebrafish. Three biological replicates per group were performed and the symbols *, **, *** indicated *p* < 0.05, 0.01, <0.001 as significant differences between DEHP treatment and control groups.

After chronic exposure to DEHP, the expression levels of immune response-related genes in gut tissues of zebrafish were measured by qRT-PCR analyses (Figure 7A). In the female zebrafish, the relative expression of gene *tlr-5* was inhibited (*p* = 0.04) by 0.48-folds in the highest DEHP exposure group, as well as the expression levels of *il-1β* decreased (*p* = 0.013–0.0001) from 0.37 to 0.69-folds in all the DEHP exposure (10–100 μg/L) groups, compared to the control group. However, in the male zebrafish, *tlr-5* and *il-1β* expressions showed no significant changes after DEHP treatment. The expression level of *il-8* was significantly increased (*p* = 0.004–0.0001) in female and male zebrafish at lower DEHP exposure groups (10 and 33 μg/L), compared to the control group. Further, the antimicrobial-related genes (*nod2*, *lyz*, *tnf-α*, and *il-10*) revealed various responses to DEHP exposure in female and male zebrafish (Figure 7B). In the female fish, although the expression levels of *nod2*, *tnf-α*, and *il-10* showed no significant difference after treatment, the expression level of critical gene *lyz* was significantly increased (*p* = 0.0008) by 1.55-fold at 100 μg/L groups, compared to control. However, in the male fish, the gene expression of *lyz* was significantly up-regulated (*p* = 0.002–0.0001) with 1.89-, 1.82-, and 1.55-fold at different DEHP groups (10–100 μg/L), as well as the *il-10* gene was significantly increased (*p* = 0.007–0.0001) with 1.86- and 2.61-fold after treating with 33 and 100 μg/L DEHP levels. Importantly, the expression levels of intestinal secretory and absorptive genes (*aqp8* and *muc2.1*), and obesity-related genes (*fgf2* and *pomca*) were significantly up-regulated in the DEHP exposed groups (Figure 7C). In the female fish, the expression level of the *aqp8* gene was up-regulated (*p* = 0.001–0.0001) with 4.53-, 3.86-, and 2.76-fold changes after treatment of 10, 33, and 100 μg/L DEHP. The *muc2.1* gene also showed significantly increased (*p* = 0.015 and 0.0004) expressions by 1.67- and 1.80-fold at 33 and 100 μg/L group, respectively, together with obesity-related genes *fgf2* (*p* = 0.012) and *pomca* (*p* = 0.0004) by 2.20- and 1.73-fold at the highest concentration group (100 μg/L), compared to control. However, in the male fish, the gene expressions showed different changes with significantly increased *aqp8* (*p* = 0.002) and *fgf2* (*p* = 0.01) with 1.96- and 1.81-fold at 10 μg/L groups, compared to the control groups. Interestingly, the mRNA level of the *pomca* gene appeared to be significantly inhibited (*p* = 0.005 and 0.002) by 0.64- and 0.43-fold at 10 and 33 μg/L DEHP treatment. These significantly changed expressions of key genes potentially indicated the effects of environmental levels of DEHP chronic exposure on female and male zebrafish *via* disrupting the intestinal functions or hemostasis.

### Relationship Among Metabolites and Critical Genes With Dominant Bacterial Genera in Zebrafish Intestines

**FIGURE 8:**
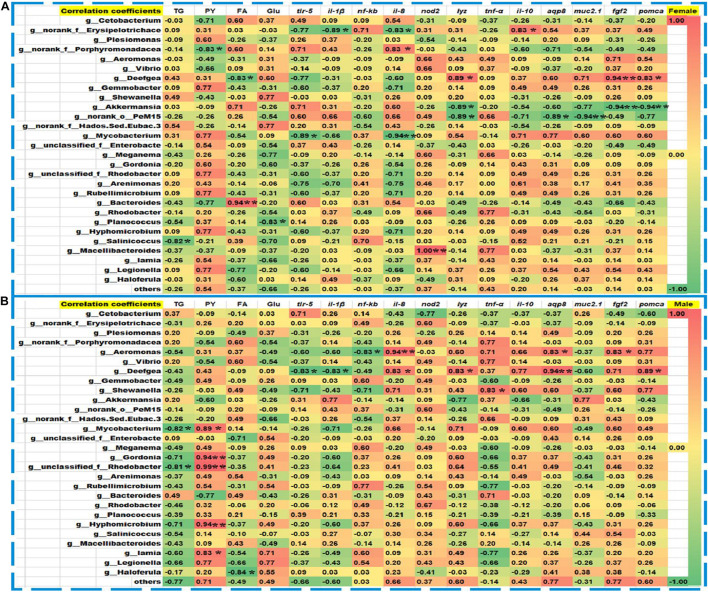
Spearman’s correlation coefficients among the abundance of intestinal microbiota, the metabolites, and the expression of intestinal functions-related genes. In the control and DEHP exposed female **(A)** and male **(B)** zebrafish, TG, PY, FA, and *tlr-5*, *lyz*, *il-8*, *tnf-α*, *aqp8*, *fgf2*, and *pomca* were sensitively related to the abundances of *Erysipelotrichaceae*, *Aeromonas*, *Deefgea*, *Mycobacterium*, *Akkermansia*, *PeM15*, and *Rhodobacter* at the genus level. The red and blue frames indicated a positive and negative correlation, respectively. Spearman’s correlation coefficients and *p* values were determined by a two-tailed test and the 95% confidence interval. The scale showed the color range of different correlation coefficients among bacteria, metabolic, and gene expression, and the symbols *, ** represented the significance levels at *p* < 0.05 and *p* < 0.01.

After DEHP exposure, Spearman’s correlation analyses were performed between the measured metabolites’ content and critical genes’ expression with the relative abundance of dominant bacteria in corresponding female and male zebrafish (Figure 8). In the control and DEHP treated female zebrafish, the contents of TG, PY, FA, and the expression levels of intestinal functional genes including *tlr-5*, *il-1β*, *il-8*, *nod2*, *lyz*, *tnf-α*, *il-10*, *aqp8*, *fgf2*, and *pomca* were significantly related to *Erysipelotrichaceae*, *Aeromonas*, *Deefgea*, *Akkermansia*, *PeM15*, *Mycobacterium*, *Bacteroides*, *Planococcus*, *Salinicoccus*, and *Macellibacteroides* at the genus level (Figure 8A). While in the male fish groups, the contents of TG, PY, FA, and the key gene expressions of *tlr-5*, *il-1β*, *il-8*, *lyz*, *tnf-α*, *aqp8*, *fgf2*, and *pomca* were significantly correlated to *Aeromonas*, *Deefgea*, *Shewanella*, *Mycobacterium*, *Gordonia*, *Rhodobacter*, *Hyphomicrobium*, *Iamia*, and *Haloferula* with positive or negative coefficients. Compared to Spearman’s analysis, Pearson’s correlation analysis also showed a similar relationship among the metabolite levels, key genes, and major genera in the exposed females and males (Supplementary Figure 9). The contents of TG, PY, FA, and the gene expressions of *trl-5*, *il-1β*, *nod2*, *tnf-α*, *il-10*, *muc2.1*, *fgf2*, and *pomca* were significantly correlated to *Erysipelotrichaceae*, *Porphyromonadaceae, Vibrio*, *Gemmobacter*, and *Akkermansia*, *Rhodobacteraceae*, *Arenimonas*, *Salinicoccus*, and *Bacteroides* in the female groups (Supplementary Figure 9A). While in the male fish, the contents of PY, FA, Glu and the expression levels of *trl-5*, *il-1β*, *il-8*, *lyz*, *tnf-α*, *aqp8*, *muc2.1*, *fgf2*, and *pomca* showed significant correlations with these genera, such as *Cetobacterium*, *Porphyromonadaceae*, *Vibrio*, *Gemmobacter, PeM15*, *Legionella*, *Gordonia*, *Mycobacterium*, *Rhodobacter*, *Bacteroides*, and *Haloferula* (Supplementary Figure 9B).

## Discussion

In recent years, elevated levels of DEHP have been reported in freshwater resources used for drinking and domestic utilities, and this could ultimately pose threats to environmental and human health (Adeogun et al., 2015; Luo et al., 2018). Previous studies on DEHP toxicity had mainly focused on the developmental and reproduction effects through various biological pathways in fish (Mu et al., 2018; Boran and Terzi, 2019). However, the eco-toxicological risks of DEHP on freshwater fish also included the disturbance of the intestinal homeostasis, especially the role of intestinal bacteria is still unclear. In this study, the toxicity of DEHP on the gut microbial community and metabolic homeostasis was investigated in male and female zebrafish after chronic exposure at environmentally relevant concentrations (10–100 μg/L). To serve the purpose, the developmental impacts, changes in the intestinal microbial community structure, the metabolites contents and the expression levels of key genes associated with immune response, and intestinal functions were highlighted. Lastly, the relationship between the changed genera, the energy metabolites, and the intestinal function-related genes was explored.

In this study, the body length and body weight of adult zebrafish were increased both in females and males, compared to control. Further, in the male fish, the critical indicators related to obesity including BMI that significantly increased in the 10 μg/L groups, and condition factor (K) was decreased in the 33 and 100 μg/L groups, compared to the control group. As reported previously, the BMI and K factor altered in zebrafish against different concentrations of DEHP mixed in food during chronic exposure (Clark et al., 2018; Buerger et al., 2019; Adamovsky et al., 2020). The significantly higher BMI in male zebrafish indicated that the chronic DEHP exposure exhibits obesity potential, albeit the organ somatic indexes (ISI, GSI, and HSI) were observed with no significant changes. Thus, we suspected that DEHP exposure might contribute to developmental effects on the whole-body weight and length instead of specific organs in zebrafish. However, it was reported that long-term exposure to DEHP with concentrations of 0.1–10 μg/L might inhibit the body growth of guppy fish under higher temperatures (Zanotelli et al., 2010). Interestingly, Huff et al. (2018) showed that low-level DEHP exposure might affect organ development by modulating the genetic responses related to fatty liver disease. Here, our results proved that the long-term DEHP exposure induced changes in zebrafish including increased body length and weight, especially sex-based significant alterations appeared in the female with higher BMI and K than the male, indicating the different ability and physiological requirements during fish growth and development from embryos to adult stages. However, the higher level of DEHP exposure possibly induces negative regulation *via* complex signaling pathways *in vivo*, which appeared as not significantly dose-dependent toxic impacts.

The changes in developmental indexes are possibly connected with the alteration of intestinal microbiota in organisms for the bacterial functions closely related to host energy extraction, lipid metabolism, endocrine functions, and immune system (Cardinelli et al., 2015; Vatsos, 2017). Previous studies also showed that the shifts in intestinal microbiota were sensitively affected by diet, gender, drugs, and environmental factors, and variations in the host’s gut microbiota can occur even at the same life-stages (Bolnick et al., 2014; Roman et al., 2019). Previously, several studies have explored the role of the gut microbial community in zebrafish’s (as a host) health during its growth and development (Roeselers et al., 2011; Stephens et al., 2016). In this study, the intestinal microbiota was subsequently investigated to explain the inner relationships of developmental indexes and homeostasis alteration in adult zebrafish. The analyses of alpha diversity (Shannon) in male fish, and phylum *Firmicutes* showed significantly different responses in both female and male fish after long-term DEHP exposure. In female zebrafish, the abundance of major phyla including *Fusobacteria*, *Bacteroidetes*, and *Actinobacteria* was decreased, while it was increased for phyla *Proteobacteria* and *Firmicutes*. In male zebrafish, the abundance of *Fusobacteria*, *Firmicutes*, and *Actinobacteria* was decreased, but for *Proteobacteri*a and *Bacteroidetes*, it was increased. *Fusobacteria* and *Bacteroidetes* were considered beneficial intestinal bacteria by providing vitamins or assist in metabolism in the gut (Xia et al., 2014). Moreover, the prevalence of *Proteobacteria* is a potential diagnostic signature of dysbiosis and other diseases (Shin et al., 2015). Here, we suspected that the disruption of those phyla was probably related to the abnormal development in female and male zebrafish associated with DEHP chronic treatment. Recently, Adamovsky et al. (2020) observed the shift of microbiome composition with the increase in abundance of phyla *Fusobacteria*, *Bacteroidetes*, and *Verrucomicrobia* in both male and female zebrafish after 2 months of DEHP exposure, and this study further showed direct effects of DEHP on several microbial metabolites of the immune and intercellular communication system by Predicted Relative Metabolic Turnover (PRMT) model. We suspected that the varying changes in major phyla such as *Fusobacteria* and *Verrucomicrobia* in Adamovsky’s and our study may be due to the difference in exposure time and life stages of zebrafish. Moreover, the composition of gut microbiota varied during zebrafish development from larvae to adult and between individuals even in the same tank, especially the distinction of environmental factors such as water source and quality, pathogen invasion, and different chemicals’ treatment (Yan et al., 2012; Stephens et al., 2016; Yang et al., 2017).

At the genus level, the abundance of major bacteria such as *Aeromonas*, *Deefgea*, *Shewanella*, and *Mycobacterium* was increased in the female and male fish. However, the abundance of *Cetobacterium*, *Akkermansia*, *Bacteroides*, *Rhodobacter*, *Planococcus*, and *Salinicoccus* were decreased in female and male fish, indicating the negative effects of DEHP on zebrafish intestinal homeostasis. Notably, the *Plesiomonas, g_norank_f_Porphyromonadaceae*, and *g_unclassified_f_Enterobacteriaceae* genera were decreased in female fish but showed higher in male fish. In contrast, the abundance of *g_norank_f_Erysipelotrichaceae*, *Gemmobacter*, *Meganema, Rubellimicrobium*, and *Legionella* genera was increased in female fish but showed lower levels in male fish. Especially, the relative abundance of *Prevotella-7*, *Deefgea*, *PeM15*, *Halomonas*, *Akkermansia*, *Streptococcus*, *Chitinibacter*, *Roseomonas*, and *Neisseria* genera in the MaAsLin analysis combined by female and male fish were significantly associated with the environmental factor of DEHP exposure. However, the major genera of *g_norank_f_Erysipelotrichaceae*, *Aeromonas*, and *Shewanella* were significantly different in exposed females compared to male fish, indicating the similarity and difference in microbial alteration. Among those altered genera, some probiotics, such as *Cetobacterium* and *Akkermansia*, were the key members essential for organisms, but some of them, such as the genera *Shewanella* and *Mycobacterium* exhibited tendencies for human infections and antibiotic resistance (Yousfi et al., 2017; Wilkes Walburn et al., 2019). Under chronic DEHP exposure, the increased endotoxins could result in obesity, inflammation, metabolic derangements, and intestinal or other organ damages (Cani et al., 2007; Boutagy et al., 2016; Tian et al., 2019). Thus, the shift in intestinal bacterial community with the changed metabolic pathways possibly comprehensively lead to a developmental abnormality, immune responses, or intestinal function disruption in the DEHP exposed zebrafish.

Environmental pollutants affect fish’s health by influencing the gut microbiota and various metabolic pathways, especially on the subsequent energy transformation, and possible host obesity (Sommer et al., 2017; Meng et al., 2018; Kindleysides et al., 2019). In this study, the intestinal microbiota explored by COG and KEGG functional analysis showed similar metabolism pathways but with different relative abundances. In detail, the pathways of lipid transport or metabolism, cell motility, and catabolism were activated in female and male fish by DEHP treatment. Especially, the metabolic pathways at the second level involved the xenobiotics biodegradation, metabolism of other amino acids and lipid metabolism signaling pathways were stimulated. Scientific evidence supported the idea that developmental indexes and metabolites are strongly related to the changes in both the function and composition of gut microbiota, which exerted an essential role in modulating energy metabolism (Avolio et al., 2019; Cerdó et al., 2019). Thus, the critical pathways of metabolism in zebrafish were significantly changed, which may explain the reason for the developmental abnormality and intestinal homeostasis after chronic DEHP exposure.

The intestine interface acts as the major barrier between organisms and the external environment, and it hosts an enormous number of microorganisms whose composition affects the epithelial functions and the intestinal immune system (La Fata et al., 2018). Evidence showed that host-microbe interactions shaped the gastrointestinal environment as well as microbiota, which benefited the development and differentiation of gut tissues (Kaiko and Stappenbeck, 2014). Notably, enteral nutrition and multiple pathologies are associated with the disruption of gut microbes after exposure to environmental pollutants. In this study, the intestines extracted from exposed fish appeared with no obvious changes in villus height, except the female in control showed lower height than corresponding male intestines. However, the villus width of male fish and muscularis thickness of female fish were significantly reduced by DEHP exposure. Importantly, the percentage of goblet cells vs. total cells per villus in intestinal tissues was significantly inhibited in the DEHP exposed female and male zebrafish. Here, our results indicated that chronic exposure to DEHP was harmful to intestinal stability by disturbing the villi and goblet cell indexes, especially in male fish. Here, the alteration of intestine entities is possibly attributed to chronic DEHP exposure, which is accompanied by other factors that can disturb the intestinal homeostasis in zebrafish, thus the subsequent metabolic functions are necessary to be explored.

Moreover, healthy microbial communities are important for the human metabolome and especially involved in the energy harvest and the regulation of body systems, and dysbiosis has been implicated in many disorders, including obesity and inflammation (Van Treuren and Dodd, 2019). Among various gut microbiota metabolites, the TG, Glu, short-chain fatty acids (SCFAs), and triglyceride triacetin have received attention for their important roles in maintaining the intestinal mucosa, metabolic substrates, and the recovery of the disease (Lynch et al., 1994; Michel et al., 2016; Feng et al., 2018; Hernandez et al., 2019). In this study, the levels of TG, PY, FA, and Glu metabolites were significantly increased in female and male zebrafish intestines, indicating the intestinal disruptions affected the intestinal mucosa in DEHP exposure groups. Our findings are consistent with the recent report on intestinal functions and lipid metabolism, implying that the intestinal networks may drive phthalate-mediated obesity in zebrafish (Buerger et al., 2019). Moreover, the metabolite levels were significantly increased in female intestines than that in the male from the same treatment groups, which may be related to the different microbial composition and physiological behaviors in both sex fish. Therefore, consistent with the changes of intestinal bacteria and related metabolic pathways, these results indicated that the potential effects of DEHP on zebrafish intestinal homeostasis.

The microbiota plays critical roles not only in intestinal development but also in the regulation of immune response and intestinal functions with high sensitivity toward environmental pollutants (Maranduba et al., 2015). In fish, the stimulation of different cytokines including *tlr-5*, *il-1β*, *nf-κb*, and *il-8*, *nod2*, *lyz*, *tnf-α*, and *il-10*, play important roles in bacterial defense and immune response, while the intestinal secretory and absorptive, obesity-related genes *aqp8*, *muc2.1*, *fgf2*, and *pomca* are involved in regulation of the intestinal diseases and digestion functions (Gomez and Balcazar, 2008; Dotti et al., 2017; Udayangani et al., 2017; Arias-Jayo et al., 2018; Montalbano et al., 2018). In this study, long-term exposure to DEHP potentially altered the expressions of genes *il-8*, *lyz*, and *il-10* related to the immune response and bacterial handling in male zebrafish, but significantly inhibited the mRNA level of *tlr-5* and *il-1β* genes in female zebrafish, especially in the highest-level exposure group (100 μg/L). The expression level of the key genes indicated the activated immune responses of zebrafish, which closely interacted with the intestinal homeostasis and microbial disruptions (Udayangani et al., 2017; Jia et al., 2021). Adamovsky et al. (2020) reported the shift of gut microbiome composition contributed to the adverse effects of DEHP on the host by altering metabolites sensed by both intestinal and immune T-helper cells. Notably, in our study, the key genes related to metabolic and intestinal functions, such as *aqp8*, *muc2.1*, *fgf2*, and *pomca* appeared to be sensitively induced in DEHP treated zebrafish, especially the expression of *pomca* was significantly down-regulated in the male zebrafish at 10–33 μg/L of DEHP exposure, indicating the different responses between male and female fish. Recently, Yang et al. (2019) reported that early life DEHP exposure altered the gut microbiota of newborns and changed their immune responses in later life. Here, the expression of key genes indicated different immune responses and intestinal function disruptions between male and female zebrafish by DEHP exposure, which may connect with various alterations of intestinal bacteria.

The intestinal metabolic and inflammation process can trigger the secretion of molecules in the host, which are required for the maintenance of the intestinal barrier function, a viscoelastic protective layer composed of mucins secreted by goblet cells (Arias-Jayo et al., 2018). From the correlation analysis, we found that the major altered bacterial genera, including *g_norank_f_Erysipelotrichaceae*, *g_norank_f_ Porphyromonadaceae*, *Aeromonas*, *Deefgea*, *Shewanella*, *Akkermansia*, *PeM15*, *g_unclassified_f_Rhodobacteraceae*, *Mycobacterium*, *Gordonia*, and *Bacteroides* were closely related to metabolite contents, immune and functionally related genes, which may affect zebrafish development. Among those major changed bacteria, the *Akkermansia* and *g_norank_f_Erysipelotrichaceae*, *Bacteroides* and *Vibrio*, were considered to regulate the bacterial functions in zebrafish or humans, which were critical for the organism’s obesity, intestinal homeostasis, and defense against pollutants and potential diseases (Wexler, 2007; Kaakoush, 2015; Derrien et al., 2017; Nag et al., 2018). Considered with the genera activated in response to DEHP exposure, the abundance changes of *Deefgea*, *PeM15* and *Akkermansia* were highly suspected bacterial bio-markers to label the intestinal disorders, which are induced by the environmental pressures. Taken all together, this study revealed that long-term exposure to DEHP induced developmental effects *via* increasing the body length and weight of zebrafish, disrupted the abundance of intestinal bacteria at phylum and genus levels, and impaired homeostasis with inflammation responses and intestinal functions, especially the differences in female and male fish. Importantly, the abundance of intestinal bacteria in female zebrafish showed a close relationship to gene expression of immune and bacterial response, but that in the male zebrafish showed higher correlation frequencies to metabolites content and gene expression of intestinal functions.

With the evidence of DEHP toxicity at environmental levels in terms of disturbed intestinal microbiota and the related metabolic functions in freshwater fish, we suspected that DEHP might increase the ecological risks for organism’s growth and development or metabolic diseases. However, there are still important questions that need to be explored to understand further the toxicity of low-level DEHP exposure, such as the content of DEHP uptake by fish in intestines and other tissues, and how the perturbation of the intestinal microbial community affects the gut development and responses associated with potential signaling pathways. Additionally, various gut bacteria at the genus level changed, albeit without statistical significance compared to control, which is a limitation to draw a concrete conclusion on the relationship between DEHP exposure and organism’s physiological responses. Thus, the bigger sample size of zebrafish intestines and application of the up-to-date software/databases were highly recommended in further researches, especially for the analysis of microbial community to understand whether the difference existed between individuals even in the same groups. We observed sex-dependent differences of microbiota, metabolites and their functions, especially exacerbated effects in female fish during development and growth compared to male fish, which should be taken into consideration for future relevant studies.

## Conclusion

In conclusion, chronic exposure to DEHP with low concentrations induced significant effects on fish development through changing body weight and length and altering the BMI of male fish. Consistently, the gut bacterial community as the more sensitive indicator showed different alterations between female and male zebrafish by DEHP exposure, especially the major phylum of *Firmicutes*, and the key genera of *Prevotella-7*, *Deefgea*, *PeM15*, *Halomonas*, and *Akkermansia*, sensitively responded to the environmental factor. Further, the KEGG analysis revealed DEHP exposure mediated the abundance of bacterial metabolic function pathways in zebrafish intestines. Regarding the disorder of metabolic homeostasis, the immune response, and intestinal functions, the female and male fish were significantly related to intestinal bacteria of *Aeromonas*, *Deefgea*, *Akkermansia*, *PeM15*, *Mycobacterium*, *Rhodobacter*, and *Bacteroides* with positive or negative correlation coefficients, which indicated the possible different responses to DEHP exposure. Therefore, the comprehensive toxicity and the intestinal hemostasis risks of DEHP on female and male zebrafish after chronic exposure cannot be ignored even at the low concentrations, where more attention should be paid to the functions of the sensitively disrupted intestinal bacterial community.

## Data Availability Statement

The datasets presented in this study can be found in online repositories. The names of the repository/repositories and accession number(s) can be found in the article/Supplementary Material.

## Ethics Statement

The animal study was reviewed and approved by Zebrafish experiments were performed according to the “Guide for the Care and Use of Laboratory Animals” (Eighth Edition, 2011. ILARCLS, National Research Council, Washington, DC, United States). The animal protocol was approved by the Animal Care and Use Committee of Chongqing in China and by the Institutional Animal Care and Use Committee of Chongqing Institute of Green and Intelligent Technology, Chinese Academy of Sciences (Approval ID: ZKCQY0063). Written informed consent was obtained from the owners for the participation of their animals in this study.

## Author Contributions

P-PJ: data curation, formal analysis, investigation, writing–original draft, methodology, validation, visualization, and funding acquisition. G-YX: data curation, formal analysis, and investigation. MJ: data curation, investigation, writing–original draft, resources, and software. YW: data curation and investigation. Y-BM: methodology, validation, and visualization. D-SP: conceptualization, funding acquisition, project administration, and writing–review and editing. All authors contributed to the article and approved the submitted version.

## Conflict of Interest

The authors declare that the research was conducted in the absence of any commercial or financial relationships that could be construed as a potential conflict of interest.

## Publisher’s Note

All claims expressed in this article are solely those of the authors and do not necessarily represent those of their affiliated organizations, or those of the publisher, the editors and the reviewers. Any product that may be evaluated in this article, or claim that may be made by its manufacturer, is not guaranteed or endorsed by the publisher.

## Footnotes

1
www.njjcbio.com

2
http://www.drive5.com/uparse/

3
http://www.arb-silva.de

4
http://www.genome.jp/kegg/

5
https://www.ncbi.nlm.nih.gov/tools/primer-blast/
