# Supplementary material for: Disruption of Intestinal Homeostasis Through Altered Responses of the Microbial Community, Energy Metabolites, and Immune System in Zebrafish After Chronic Exposure to DEHP
Source: Front Microbiol. 2021 Oct 4;12:729530. doi: 10.3389/fmicb.2021.729530 (PMC8524448; doi:10.3389/fmicb.2021.729530)
Supplement: Supplementary file 1 [file Data_Sheet_1.docx]

**Supplementary Information**

**Disruption of intestinal homeostasis through altered responses of the microbial community, energy metabolites, and immune system in zebrafish after chronic exposure to DEHP**

Pan-Pan Jia^1,2^, Muhammad Junaid^3^, Guang-Yuan Xin^2^, Yan Wang^2^, Yan-Bo Ma^2^, De-Sheng Pei^1^*
^1^School of Public Health and Management, Chongqing Medical University, Chongqing 400016, China
^2^Chongqing Institute of Green and Intelligent Technology, Chinese Academy of Sciences, Chongqing, 400714, China
^3^Joint Laboratory of Guangdong Province and Hong Kong Region on Marine Bioresource Conservation and Exploitation, College of Marine Sciences, South China Agricultural University, Guangzhou, 510642, China
*Corresponding author. E-mail: deshengpei@gmail.com (D.S.P)

**Text S1. The methods of total DNA extraction, PCR amplification, 16S rRNA sequencing of zebrafish intestinal microbiota**

At first, the total genomic DNA of intestinal samples was extracted and purified with FastDNA^®^ SPIN Kit for Soil (Mpbio, USA). The concentration and purity of extracted DNA samples were assessed by a NanoDrop 2000 Spectrophotometer (Thermo Scientific, USA), and the integrity of DNA was checked by 1% agarose gel electrophoresis. The concentration of extracted DNA was assessed by a NanoDrop ND-2000 (Thermo Fisher Scientific, USA). Amplicons for sequencing were amplified as previously described ([Jia et al., 2019](#_ENREF_1)). Briefly, extracted DNA was first amplified with high-fidelity Taq polymerase (Invitrogen, USA). Then equal quantities of three PCR reactions per sample were pooled, purified with the QIAquick PCR Purification Kit (Qiagen, Valencia, CA). The PCR amplification of 16S rRNA gene was performed as follows: initial denaturation at 95 ^o^C for 3 min, followed by 27 cycles of denaturing at 95 ^o^C for 30 s, annealing at 55 ^o^C for 30 s, and extension at 72 ^o^C for 45 s, and single extension at 72 ^o^C for 10 min, and end at 4  ^o^C by an ABI GeneAmp® 9700 PCR thermocycler (ABI, CA, USA). Finally, the purified amplicons were pooled in equimolar and paired-end sequenced on an Illumina MiSeq PE300 platform (Illumina, San Diego, USA) according to the standard protocols by Majorbio Bio-Pharm Technology Co. Ltd. (Shanghai, China). Raw reads were sorted and can be analyzed using the platform of the company Majorbio (<http://www.i-sanger.com/>).

**Text S2. The measures of TG, PY, FA, and Glu levels**

For the measurement of the TG content, the gut tissues of adult zebrafish were weighted, and the homogenization medium was added by weight (g): volume (mL) 1: 9. Then, the tissues were mechanically ground on ice and then centrifuged for 10 min at 2500 rpm. Finally, the supernatant liquid, standards, and ddH_2_O were mixed in the kits with the working medium at 37^o^C for 10 min. After incubation, the OD value was measured at 510 nm wavelengths.

For the measurement of the PY content, the standards and ddH_2_O as the blank, and the supernatant liquid samples were mixed with working agent II in the kits at 37^o^C for 10 min, and agent III for 5 min at room temperature. After incubation, the OD value was measured at 505 nm wavelengths.

For the measurement of the FA content, the standards and ddH_2_O as the blank, and the supernatant liquid samples were mixed in the kits with the working agent at 37^o^C for 10 min, and then the OD value was measured at 546 nm wavelengths as the A1 data. After incubation agent II for 5 min at room temperature, the OD value was measured at 546 nm wavelengths as the A2 data, and ΔA= A2-A1.

For measurement of the glucose (Glu) content, the standards and ddH_2_O as the blank and the supernatant liquid samples were mixed in the kits with the working agent at 37^o^C for 15 min, and the OD value was measured at 505 nm wavelengths.

**Table S1. The analysis software or database of 16s rRNA sequencing used in this study**

| **Analysis software/database** | **Version** | **Use** | **Links** |
| --- | --- | --- | --- |
| Flash | 1.2.11 | Pair-end sequencing reads merged | https://ccb.jhu.edu/software/FLASH/index.shtml |
| Qiime | 1.9.1 | Taxonomies, Beta diversity, distance calculations | http://qiime.org/install/index.html |
| Uparse | 7.0.1090 | OUT Clustering | http://www.drive5.com/uparse/ |
| RDP Classifier | 2.11 | Sequence classification annotation | https://sourceforge.net/projects/rdp-classifier/ |
| Usearch | 7 | OUT Statistics | http://www.drive5.com/usearch/ |
| Mothur | 1.30.2 | alpha diversity | https://www.mothur.org/wiki/Download_mothur |
| PICRUSt | 1.1.0 | KEGG、COG、Pfam Functional prediction | http://picrust.github.io/picrust/ |
| Mega | 7 | Evolutionary Tree Analysis | https://www.megasoftware.net/ |
| SILVA | 138 | rRNA database | https://www.arb-silva.de/ |
| RDP | 11.5 | rRNA database | http://rdp.cme.msu.edu/ |
| GreenGenes | 135 | rRNA database | http://greengenes.secondgenome.com/ |
| FunGene | 9.6 | Functional Gene Database | http://www.fungene-db.fr/ |
| HPB | -- | human pathogens database | https://www.cerl.org/resources/hpb/content |
| Tax4fun | 0.3.1 | Tax4Fun Functional prediction | http://tax4fun.gobics.de/ |
| MAFFT | 7.2 | Multiple sequence alignment | https://mafft.cbrc.jp/alignment/software/ |
| IQ-TREE | 1.6.8 | Making evolutionary trees | http://www.iqtree.org/ |
| Fastp | 0.19.6 | Quality Control | https://github.com/OpenGene/fastp |
| PICRUSt2 | 2.2.0 | KEGG orthologys (KO), EC, COG, MetaCyc metabolic pathways | https://github.com/picrust/picrust2/ |

**Table S2. The developmental indexes of zebrafish after chronic exposure to DEHP**

| **Groups** | **Body length (cm)** | **Bodyweight (g)** | **Body mass index (BMI, mg/cm^2^)** | **Condition factor (K, g*100/cm^3^)** | **Intestinal somatic index (ISI, %)** | **Gonadal somatic index (GSI, %)** | **Hepatic somatic index (HSI, %)** |
| --- | --- | --- | --- | --- | --- | --- | --- |
| 0-F | 2.881±0.027 | 0.201±0.005 | 24.136±0.311*** | 0.840±0.013*** | 4.706±0.249*** | 9.941±0.584 | 1.818±0.201** |
| 10-F | 3.056±0.029*** | 0.233±0.005***/** | 25.074±0.373*** | 0.820±0.017** | 5.300±0.227*** | 10.252±0.726 | 1.856±0.139*** |
| 33-F | 2.956±0.028* | 0.213±0.005 | 24.304±0.332*** | 0.825±0.015*** | 5.136±0.251*** | 8.705±0.567 | 1.944±0.146*** |
| 100-F | 2.895±0.031** | 0.213±0.005* | 25.137±0.425*** | 0.859±0.018*** | 4.591±0.235*** | 11.095±0.709 | 1.969±0.138*** |
| 0-M | 2.951±0.022 | 0.193±0.004 | 22.144±0.249 | 0.752±0.010 | 3.334±0.215 | 0.998±0.140 | 1.118±0.150 |
| 10-M | 3.021±0.023* | 0.210±0.005** | 23.028±0.274* | 0.762±0.010 | 3.584±0.161 | 1.082±0.112 | 1.128±0.144 |
| 33-M | 3.068±0.036** | 0.207±0.006* | 21.968±0.247 | 0.719±0.012* | 3.419±0.178 | 1.037±0.152 | 1.179±0.140 |
| 100-M | 3.026±0.025* | 0.196±0.005 | 21.655±0.275 | 0.718±0.011* | 3.011±0.141 | 1.262±0.145 | 1.014±0.123 |

Note: The data were presented as the mean ± standard error (SEM) of 12 pairs per tank and 3 replicate tanks (totally n=36) of zebrafish samples in each group, and the symbols black *, **, and *** indicated the significant changes between the exposure groups and control with *p* < 0.05, 0.01, and 0.001, respectively. The symbol *, **, and *** indicated the significant changes between the female and male in the same groups, except the GSI with no comparison.

**Table S3. The richness and diversity indexes of zebrafish intestinal microbiota^a^**

| **Groups** | **OTUs number** | **Shannon** | **Simpson** | **Ace** | **Chao** | **Coverage (%)** |
| --- | --- | --- | --- | --- | --- | --- |
| F-0 | 206.33±28.17 | 2.38±0.24 | 0.20±0.04 | 252.32±12.10 | 246.18±19.03 | 99.94±0.00 |
| F-100 | 231.33±31.02 | 2.35±0.36 | 0.21±0.04 | 261.28±20.81 | 263.55±17.09 | 99.94±0.01 |
| M-0 | 171.00±10.97 | 1.87±0.29 | 0.30±0.07 | 218.87±14.24 | 209.74±18.86 | 99.93±0.01 |
| M-100 | 182.67±20.50 | 2.15±0.03* | 0.23±0.03 | 211.41±8.90 | 207.91±10.54 | 99.95±0.01 |

^a^All data were presented as the mean ± standard error (SEM) of three replicates in each group, and the symbol * implied the significant changes with *p*<0.05 between the DEHP exposed group and control.

**Table S4. The changes of zebrafish gut microbiota at genus level^a^**

| **OTU ID** | | **F-0** | **F-100** |  | **M-0** | **M-100** |  |
| --- | --- | --- | --- | --- | --- | --- | --- |
|  |  | Percentage (%) | Percentage (%) | Fold  change | Percentage (%) | Percentage (%) | Fold  change |
| 1 | *p__Fusobacteria; g__Cetobacterium* | 35.23 | 27.71 | 0.79 ↓ | 47.81 | 41.85 | 0.88 ↓ |
| 2 | *p__Firmicutes; g__norank_f__Erysipelotrichaceae* | 17.15 | 27.39 * | 1.60 ↑ | 20.06 | 13.68 | 0.68 ↓ |
| 3 | *p__Proteobacteria; g__Plesiomonas* | 11.19 | 7.33 | 0.65 ↓ | 4.58 | 5.99 | 1.31 ↑ |
| 4 | *p__Bacteroidetes; g__norank_f__Porphyromonadaceae* | 5.85 | 4.17 | 0.71 ↓ | 6.33 | 10.62 | 1.68 ↑ |
| 5 | *p__Proteobacteria; g__Aeromonas* | 4.28 | 5.51* | 1.29 ↑ | 4.67 | 7.56 | 1.62 ↑ |
| 6 | *p__Proteobacteria; g__Vibrio* | 1.89 | 1.57 | 0.83 ↓ | 4.57 | 4.49 | 0.98 ↓ |
| 7 | *p__Proteobacteria; g__Deefgea* | 0.67 | 4.47 | 6.73 ↑ | 0.66 | 2.98 * | 4.53 ↑ |
| 8 | *p__Proteobacteria; g__Gemmobacter* | 3.28 | 4.10 | 1.25 ↑ | 0.70 | 0.36 | 0.52 ↓ |
| 9 | *p__Proteobacteria; g__Shewanella* | 0.72 | 0.84* | 1.17 ↑ | 1.36 | 2.76 | 2.03 ↑ |
| 10 | *p__Verrucomicrobia; g__Akkermansia* | 1.69 | 0.67 | 0.40 ↓ | 2.22 | 0.45 | 0.20 ↓ |
| 11 | *p__Actinobacteria; g__norank_o__PeM15* | 3.07 | 0.28 | 0.09 ↓ | 1.49 | 0.02 | 0.02 ↓ |
| 12 | *p__Fusobacteria; g__norank_f__Hados.Sed.Eubac.3* | 0.62 | 0.23 | 0.37 ↓ | 0.99 | 1.76 | 1.79 ↑ |
| 13 | *p__Actinobacteria; g__Mycobacterium* | 0.48 | 2.32 | 4.85 ↑ | 0.07 | 0.31 | 4.37 ↑ |
| 14 | *p__Proteobacteria; g__unclassified_f__Enterobacteriaceae* | 0.84 | 0.51 | 0.60 ↓ | 0.33 | 0.43 | 1.30 ↑ |
| 15 | *p__Proteobacteria; g__Meganema* | 0.67 | 1.17 | 1.76 ↑ | 0.18 | 0.04 | 0.23 ↓ |
| 16 | *p__Actinobacteria; g__Gordonia* | 0.55 | 0.56 | 1.01 ↑ | 0.09 | 0.61 | 6.71 ↑ |
| 17 | *p__Proteobacteria; g__unclassified_f__Rhodobacteraceae* | 0.26 | 0.90 | 3.53 ↑ | 0.08 | 0.41 | 5.10 ↑ |
| 18 | *p__Proteobacteria; g__Arenimonas* | 0.59 | 0.86 | 1.46 ↑ | 0.08 | 0.07 | 0.87 ↓ |
| 19 | *p__Proteobacteria; g__Rubellimicrobium* | 0.62 | 0.82 | 1.32 ↑ | 0.11 | 0.04 | 0.33 ↓ |
| 20 | *p__Bacteroidetes; g__Bacteroides* | 0.34 | 0.20 | 0.58 ↓ | 0.54 | 0.46 | 0.86 ↓ |
| 21 | *p__Proteobacteria; g__Rhodobacter* | 0.73 | 0.31 | 0.42 ↓ | 0.36 | 0.02 | 0.06 ↓ |
| 22 | *p__Firmicutes; g__Planococcus* | 1.27 | 0.01 | 0.01 ↓ | 0.01 | 0.01 | 0.55 ↓ |
| 23 | *p__Proteobacteria; g__Hyphomicrobium* | 0.43 | 0.69 | 1.59 ↑ | 0.03 | 0.06 | 2.02 ↑ |
| 24 | *p__Firmicutes; g__Salinicoccus* | 1.08 | 0.00 | 0.00 ↓ | 0.00 | 0.00 | 0.17 ↓ |
| 25 | *p__Bacteroidetes;g__Macellibacteroides* | 0.07 | 0.12 | 1.77 ↑ | 0.20 | 0.54 | 2.76 ↑ |
| 26 | *p__Actinobacteria; g__Iamia* | 0.33 | 0.45 | 1.36 ↑ | 0.05 | 0.05 | 1.01 ↑ |
| 27 | *p__Proteobacteria; g__Legionella* | 0.14 | 0.56 | 3.86 ↑ | 0.04 | 0.05 | 1.08 ↑ |
| 28 | *p__Verrucomicrobia; g__Haloferula* | 0.02 | 0.06 | 3.28 ↑ | 0.03 | 0.34 | 13.08 ↑ |
| 29 | *others* | 5.94 | 6.16 | 1.04 ↑ | 2.36 | 4.05 | 1.71 ↑ |

**^a^**The data showed the richness of various genera with the average percentage (%) of replicates in each group, and the fold change of DEHP exposed female and male zebrafish was calculated by average percentages *vs* that of control. The black color * stands for the significant difference *p*<0.05 between exposed fish and control, and the red color * stands for the significant difference *p*<0.05 between female and male fish.


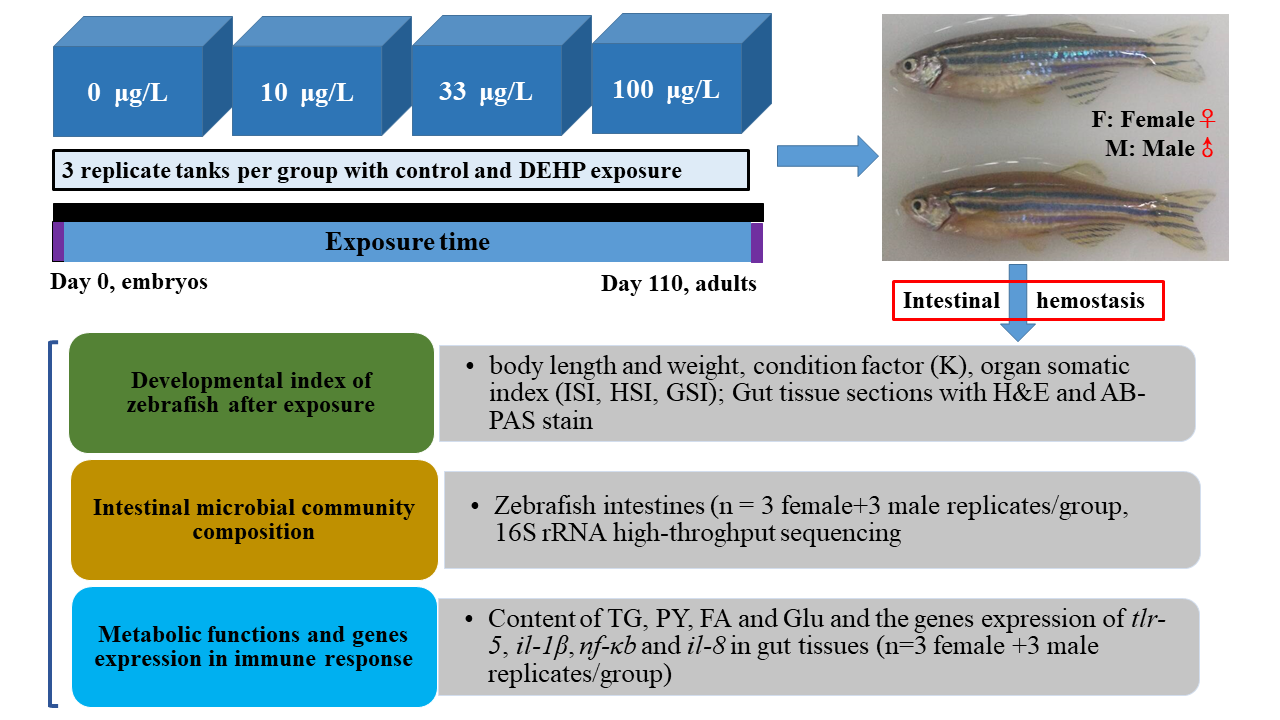


**Fig. S1. The design of DEHP chronic exposure experiment on zebrafish from embryos to adults.** 36 pairs of zebrafish were collected for the developmental indexes, and the three replicates were carried out (n=3) for female and male samples in each group.





**Fig. S2. The mortality at 24 and 50 dpf of zebrafish with a daily record from embryos to adults.** After 50 days, the zebrafish were stable with survival ability based on the no death in one week. The data showed the 24 dpf (15.09-27.65%) and 50 dpf (17.84-29.22%) mortality of zebrafish in different groups with three replicate tanks include the female and male without significant changes compared to the control group by t-test analysis and *p*-value of 0.05.


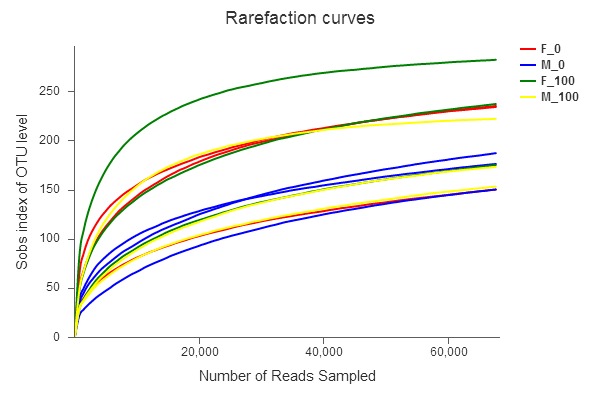


**Fig. S3. The rarefaction curve of sequences based on the > 97% similarity level.** Different colors showed different groups with three replicate samples, and the F-0 and M-0 indicated the female and male fish in the control group.

**
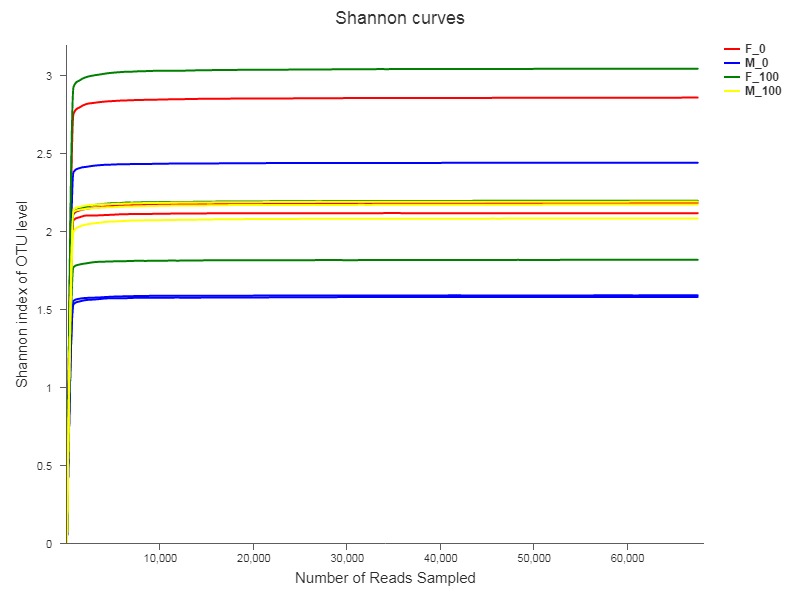
**

**Fig. S4. The Shannon rarefaction curve of sequences on OTUs level (>97% similarity level).** Different colors showed different groups with three replicate samples, and the F-0 and M-0 indicated the female and male fish in the control group.

**
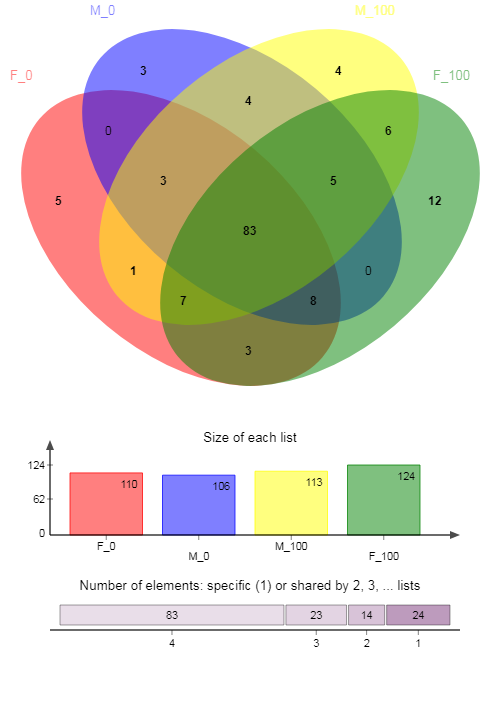
**

**Fig. S5. The Venn chart in the family level showed the similarity of each group by detecting the specific or shared family members.** The shared family member by the four groups was 83, and a total of 24 families were specific from those groups. The male fish showed high similarity in the control and DEHP groups, while the female fish in the F-100 group had more special families.


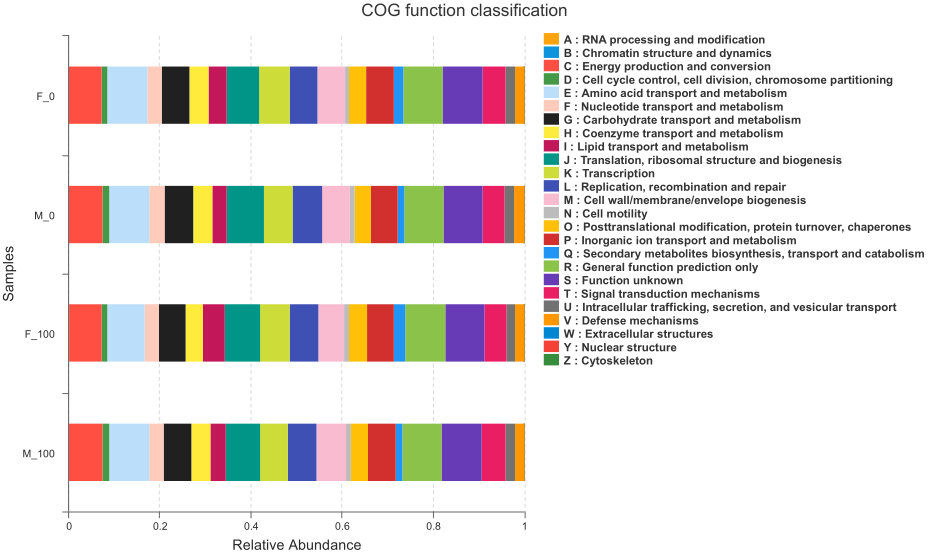


**Fig. S6. The abundance of mechanism pathways of zebrafish bacterial functions by COG analysis**. The various mechanism pathways with different relative abundance were compared between the female and male zebrafish, as well as the DEHP exposure and control groups.

**
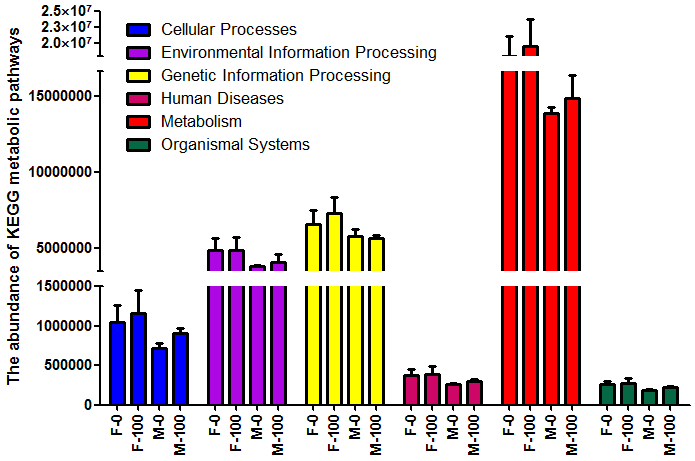
**

**Fig. S7. The abundance of mechanism pathways of zebrafish bacterial functions by KEGG analysis**. The different levels of mechanism pathways were calculated from the MiSeq Illumina platform, and the metabolism pathway showed a higher level among the most critical pathways for body health.

**
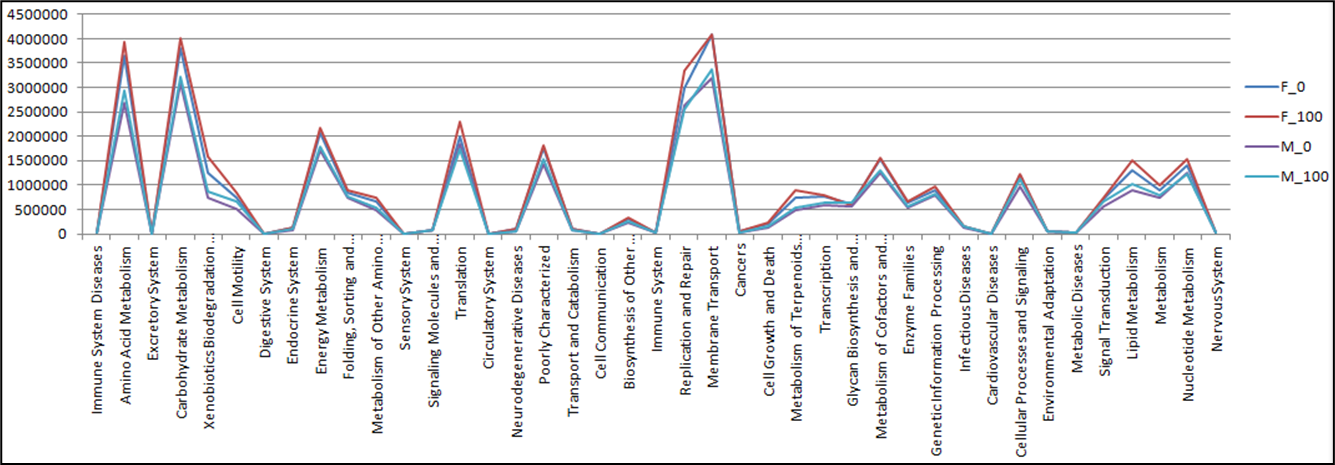
**

**Fig. S8. The relative abundance of metabolic pathways at the second level.** The values were represented with the average abundance of three replicate samples, and the metabolic pathways showed different levels in female and male zebrafish in DEHP exposure and control groups.

**Fig. S9. Pearson’s correlation coefficients among the abundance of changed genera, the contents of metabolites, and the expression levels of intestinal functions-related genes**. In the DEHP exposed female (A) and male (B) groups, PY, FA, and *tlr-5*, *lyz*, *tnf-α*, *aqp8*, *fgf2*, *pomca* were sensitively related to *Cetobacterium*, *Deefgea*, *Gemmobacter*, *Akkermansia*, *PeM15*, and *Rhodobacter* changed abundance. The scale shown on the left is the color range of different correlation coefficient values among bacteria, metabolic, and gene expression. The symbols *, ** represents the significance levels at *p*<0.05 and *p*<0.01

**References**

Jia, P.P., Sun, T., Junaid, M., Xiong, Y.H., Wang, Y.Q., Liu, L., Pu, S.Y., Pei, D.S., 2019. Chronic exposure to graphene oxide (GO) induced inflammation and differentially disturbed the intestinal microbiota in zebrafish. Environ Sci-Nano 6, 2452-2469.
